# Advancing standardization of diagnostics and antimicrobial susceptibility testing for pathogenic mycoplasmas of livestock origin: insights from the MyMIC network

**DOI:** 10.1186/s12917-025-05154-4

**Published:** 2025-12-29

**Authors:** Maryne Jaÿ, Sara M. Klose, Marco Bottinelli, Tiina Autio, Claire A.M. Becker, Jade Bokma, Cécile Boland, Filip Boyen, Salvatore Catania, Katarzyna Dudek, Emma Hurri, Anneke Feberwee, Miklós Gyuranecz, Inna Lysnyansky, Lucía Manso-Silván, Franca Möller Palau-Ribes, Ana S. Ramirez, Anne Ridley, Joachim Spergser, Paola K. Vaz, Nadeeka Wawegama, Jeanine Wiegel, Annet E. Heuvelink, Gudrun Overesch, Anne V. Gautier-Bouchardon, Florence Tardy

**Affiliations:** 1 Université de Lyon, UMR Mycoplasmoses Animales, ANSES, VetAgro Sup, Lyon, France; 2 Asia-Pacific Centre for Animal Health, Melbourne Veterinary School, Faculty of Science, The University of Melbourne, Parkville, VIC 3010 Australia; 3 Mycoplasma unit, WOAH reference laboratory for Avian Mycoplasmosis, Istituto Zooprofilattico Sperimentale delle Venezie, via Bovolino 1c, Buttapietra (VR), 37060 Italy; 4 Animal Health Diagnostic Unit, Laboratory and Research Division, Finnish Food Authority, Kuopio, 70210 Finland; 5 Faculty of Veterinary Medicine, Ghent University, Salisburylaan 133, Merelbeke, B- 9820 Belgium; 6 Sciensano, Veterinary Bacteriology, Ixelles, Belgium; 7 Department of Bacteriology and Bacterial Animal Diseases, National Veterinary Research Institute, Pulawy, Poland; 8 Department of Animal Health and Antimicrobial Strategies, Swedish Veterinary Agency (SVA), Uppsala, SE-751 89 Sweden; 9 Royal GD, Deventer, The Netherlands; 10 HUN-REN Veterinary Medical Research Institute, Budapest, Hungary; 11 Mycoplasma Unit, Kimron Veterinary Institute, Beit Dagan, 50250 Israel; 12 CIRAD UMR ASTRE, INRAE, Université de Montpellier, Montpellier, F- 34398 France; 13 Clinic for Birds, Reptiles, Amphibians and Fish, Justus-Liebig University Gießen , Gießen, Germany; 14 Veterinary Faculty, Unidad de Epidemiología y Medicina Preventiva, IUSA, Universidad de Las Palmas de Gran Canaria, Arucas – Las Palmas, Spain; 15 Department of Bacteriology, Animal and Plant Health Agency, Addlestone, Surrey, KT15 3NB United Kingdom; 16 University of Veterinary Medicine Vienna (Vetmeduni), Vienna, 1210 Austria; 17 Institute of Veterinary Bacteriology, University of Bern, Bern, Switzerland; 18 Laboratoire de Ploufragan-Plouzané-Niort, Unité Mycoplasmologie, Bactériologie et Antibiorésistance, ANSES, Ploufragan, France

**Keywords:** Mycoplasma, Diagnosis, Antimicrobial susceptibility testing (AST), Minimum inhibitory concentration (MIC), Livestock, Veterinary practices

## Abstract

**Background:**

Mycoplasma infections pose a significant economic burden and represent a serious health and welfare concern for the livestock sector. Their control often requires repeated antimicrobial treatments. Antimicrobial susceptibility testing (AST) procedures for veterinary mycoplasmas lack standardization. Furthermore, clinical breakpoints (CBPs) are not available to interpret AST data (i.e., Minimum Inhibitory Concentration, MIC values) and categorize isolates as susceptible, resistant, or intermediate to the different antimicrobials used in livestock, nor epidemiological cut-offs (ECOFFs), which are a prerequisite to define CBPs. In 2023, the MyMIC network - a consortium of 22 laboratories specializing in veterinary mycoplasmas- was established to support efforts in standardizing diagnostics and AST, including clinical interpretation. Its initial goals were to (i) review routine diagnostic practices in frontline laboratories and examine veterinarians’ prescribing habits and (ii) assess practices for culture, identification and AST used in expert laboratories and how these practices may affect MIC results as collected for five major livestock pathogens *(M. bovis*, *M. gallisepticum*, *M. synoviae*, *M. hyopneumoniae* and *M. hyorhinis*).

**Results:**

A first survey targeting veterinarians from the avian, porcine, and ruminant livestock sectors provided 468 complete responses from 39 countries worldwide, giving an account of current trends in the treatment and first-line diagnosis of veterinary mycoplasmoses. Macrolides, tetracyclines, pleuromutilins, florfenicol and fluoroquinolones were the most frequently administered antimicrobials, with usage varying by livestock sector. Veterinarians reported requesting diagnostic in 40–75% of clinical cases, but only one-third requested AST regularly. A separate survey within the consortium highlighted significant variability in the media and methods used by specialized laboratories, particularly for MIC determination, which relied mostly on in-house broth dilution techniques. This methodological diversity limited our ability to aggregate collected MIC data for establishing ECOFFs.

**Conclusions:**

Several concerns regarding best practices for antimicrobial treatments of mycoplasma infections may be linked to the lack of AST in frontline laboratories. Based on information collected in expert laboratories, we identified multiple sources contributing to inconsistent MIC results. The next step will be to establish consensus gold-standard AST methods tailored to specific mycoplasma-antimicrobial combinations to generate reliable MIC data for defining ECOFFs. Subsequently, the development of ready-to-use commercial MIC plates for use in frontline laboratory will support veterinarians in selecting appropriate treatments.

**Supplementary Information:**

The online version contains supplementary material available at 10.1186/s12917-025-05154-4.

## Background

Bacteria of the *Mycoplasma* genus, trivially referred to as mycoplasmas, are characterized by small and wall-less cells. Their genome has a low G + C% and is considered minimal, albeit with sufficient coding capacity to allow autonomous replication in axenic medium. The loss of several metabolic pathways in the course of evolution has resulted in fastidious bacteria relying on complex in vitro growth media. Several species are major pathogens in humans or animals [1]. For the sake of clarity and consistency with previous studies, the current List of Prokaryotic Names with Standing in Nomenclature (LPSN) recommendations [2], and recent developments [3], the “classical” taxonomy of the *Mycoplasma* genus [4, 5] is used throughout this manuscript and alternative names referring to the revised taxonomy proposed by Gupta et al. [6] are mentioned in brackets. In livestock, mycoplasma-associated diseases are common and often progress to chronic or lifelong infections (e.g., carrier states). They pose a significant economic burden and represent a health and welfare concern in avian, porcine, and ruminant livestock populations [7]. Control strategies vary; culling may be applicable to certain sectors associated with trade and requiring disease freedom, whereas vaccination –when available- and farm-level biosecurity measures are also essential. However, these control strategies face significant limitations. For instance, several of the current mycoplasma vaccines provide only partial protection or are ineffective at preventing transmission in host species such as poultry and swine [8, 9]. Consequently, the control of these insidious diseases often requires the implementation of repeated antimicrobial treatments.

Several studies have shown that suboptimal antimicrobial treatments - that fail to completely eradicate the pathogens- can result in reduced antimicrobial susceptibility and increased acquired resistance in mycoplasmas, hence hampering treatment efficacy [10, 11]. Among animal mycoplasmas, the most alarming situation currently concerns *Mycoplasma (M.) bovis [Mycoplasmopsis bovis]*, which demonstrates elevated in vitro MICs to multiple antimicrobial classes, except fluoroquinolones. Fluoroquinolone-based treatment potentially supported by low MIC values and experimental results in a *M. bovis* infection model is however not a recommended first-line option [11–13], since fluoroquinolones are considered critical for human medicine [14] and should be used as last resort antimicrobials in livestock. Of note, the intrinsic resistance of mycoplasmas is important. It precludes the use of antimicrobials that inhibit cell-wall synthesis - such as β-lactams, glycopeptides, and fosfomycin - as well as first-line antimicrobials like sulfonamides and trimethoprim. Some mycoplasmas including *Mycoplasma (M.) synoviae [Mycoplasmopsis synoviae]*,* Mycoplasma (M*.) *hyopneumoniae [Mesomycoplasma hyopneumoniae]* and *M. bovis*, are further naturally resistant to 14-membered ring macrolides such as erythromycin [10].

The appropriate choice of treatment for different mycoplasmas and host body-sites is essential. The in vivo efficacy of antimicrobials can be indirectly assessed by in vitro antimicrobial susceptibility testing (AST), but procedures used for other bacteria like disk diffusion tests are not suitable for mycoplasmas and other techniques, such as broth dilution, are not currently standardized for veterinary mycoplasmas [15]. The lack of approved guidelines and quality control strains agreed as most suitable for AST of livestock mycoplasmas affects the quality and comparability of results from different laboratories. Moreover, clinical breakpoints (CBPs) are lacking, and only a few epidemiological cut-off values (ECOFFs) are available for the accurate and harmonized interpretation of raw AST data (e.g. Minimum Inhibitory Concentration, MIC values) and categorization of isolates as susceptible, resistant or intermediate to the different antimicrobials currently in use. The combination of these deficiencies may ultimately compromise the effectiveness of treatments. Early in the diagnostic process, the challenges associated with culturing and identifying mycoplasma isolates to the species level may also contribute to discouraging first-line laboratories from performing AST. No standard medium for an optimal growth of all veterinary mycoplasmas exists due to the diversity of nutritional requirements of different species [1]. The most recent recommendations for performing AST on veterinary mycoplasma species date back to 25 years ago [16]. In contrast, human medicine has its approved guideline since 2011: “M43-A, Methods for Antimicrobial Susceptibility Testing for Human Mycoplasmas” [17], adopted by the Clinical and Laboratory Standards Institute (CLSI), a not-for-profit organization that develops laboratory standards worldwide. Unfortunately, this standard cannot be used for animal mycoplasmas because of their different tissue tropisms and nutritional requirements.

In 2023, the MyMIC network, a consortium of 22 research organizations from 18 countries working on animal mycoplasmas, was set up under the funding umbrella of the Joint Programming Initiative on Antimicrobial Resistance (JPIAMR). The ultimate goal of the MyMIC network is to establish guidelines for the standardization of culture, identification and AST –including clinical interpretation- of veterinary mycoplasmas. This paper presents the outcomes achieved to date by the MyMIC network. These include: (i) reviewing practices for the routine diagnosis of mycoplasmoses in frontline laboratories without specific expertise in mycoplasmology, (ii) examining antimicrobial prescription practices by veterinarians in different livestock sectors in case of presumptive mycoplasmosis; and (iii) assessing practices for culture, identification and AST in partner laboratories with recognized expertise in mycoplasmology.

Several MyMIC partners also provided MIC values of the main classes of antimicrobials used against key pathogenic *Mycoplasma* species, together with detailed methodologies used to generate these results. This dataset was used to assess the current level of harmonization in AST methods and the consistency of the resulting MIC distributions, as a preliminary step toward establishing ECOFFs.

## Results

### Account of current practices applied for the control of mycoplasmoses in livestock

**Fig. 1:**
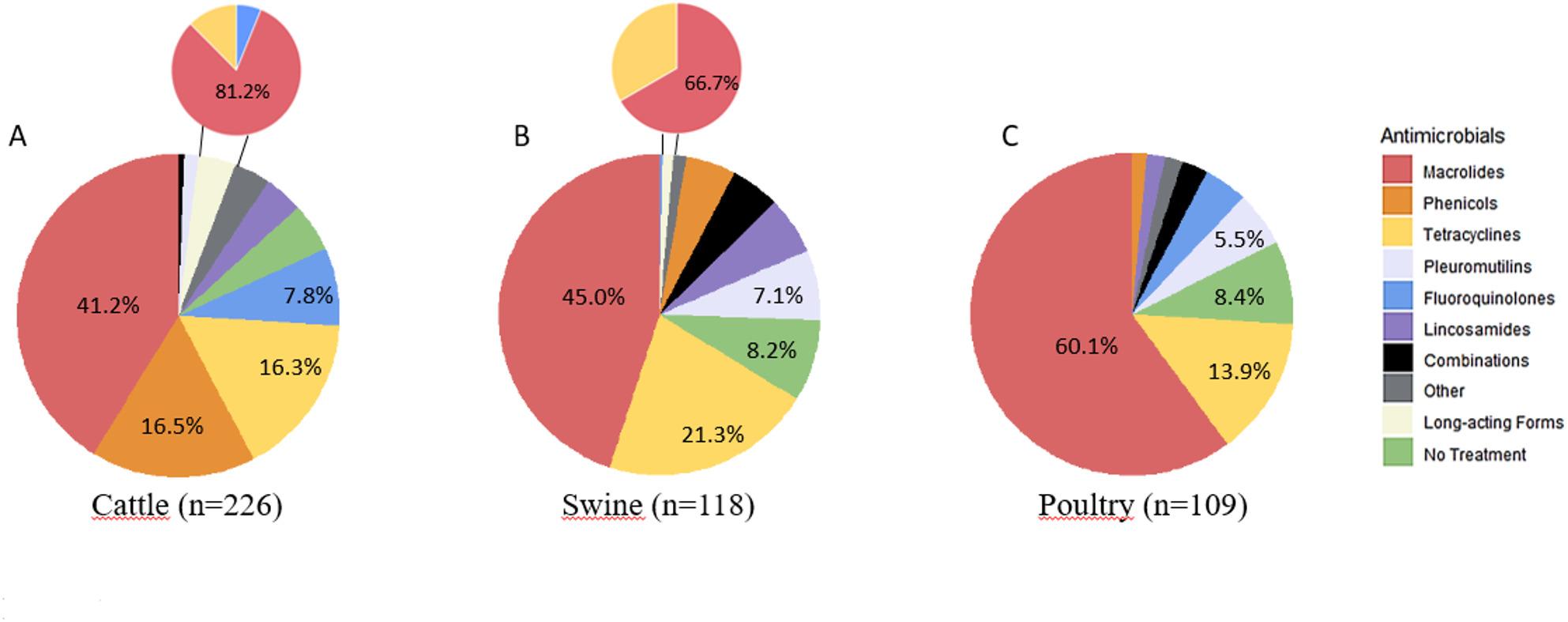
First-choice antimicrobial classes used in cattle (panel A, n=226), swine (panel B, n=118) and poultry (panel C, n=109) according to the veterinarians’ survey. The size of each distribution slice is proportional to the percentage of usage as reported by respondents. The main antimicrobial classes are indicated with their corresponding percentages. “Combinations” refer to macrolides & tetracyclines for cattle and poultry and to tetracyclines & pleuromutilins or tetracyclines & macrolides or macrolides & florfenicol for swine. “Other” refer to penicillins (both with and without beta-lactam inhibitors), trimethoprim/sulfonamides, aminoglycosides, and cephalosporins.“Long-acting forms” are detailed per antimicrobial classes and refer to tulathromycin, long-acting tetracyclines and enrofloxacin

The survey on antimicrobial use for treating mycoplasmoses resulted in 468 complete responses from volunteer veterinarians in 39 countries. Responses mainly originated from European countries (*n* = 336), but also from other regions worldwide: Africa (*n* = 30), Asia (*n* = 34), North America (*n* = 38), Central and South America (*n* = 16) and Oceania (*n* = 14). Figure 1 presents the molecules used as first-choice treatment for cattle), swine, and poultry. Overall, the main molecules used for first-choice treatment of suspected mycoplasmosis were macrolides, tetracyclines, pleuromutilins, and florfenicol. Of note a “no treatment option” was mentioned in up to 8% of clinical cases. The main molecules used in cases of first-treatment failure were the same as those used for first-choice treatment for all species, except in bovines, where fluoroquinolones became the most frequently used family (26.1%).

The survey also focused on diagnosis and AST run by first-line diagnostic laboratories. Veterinarians reported the implementation of laboratory diagnosis in approximately 40% of cattle cases, 53% of swine cases and 75% of poultry cases, with a frequency of either “always” (more than 80%) or “often” (more than 50%). Among the methods indicated in the survey, the PCR-test was the predominant technique for *Mycoplasma* species identification, employed in approximately 40% of cases across all animal species. A minority of veterinarians reported never performing laboratory diagnosis in case of suspected mycoplasmosis in cattle (10%), swine (2.5%) and poultry (1.8%). The results of the survey highlighted challenges in the implementation of AST in the routine workflow of first-line laboratories, with 28%, 46%, and 30% of cattle, swine, and poultry veterinarians, respectively, reporting that they never request AST, versus, respectively 23%, 30% and 37% reporting to have AST performed always (more than 80% of clinical cases) or often (more than 50% of clinical cases).

### Inventory of current procedures implemented for Mycoplasma spp. culture and identification in partner laboratories

**Fig. 2:**
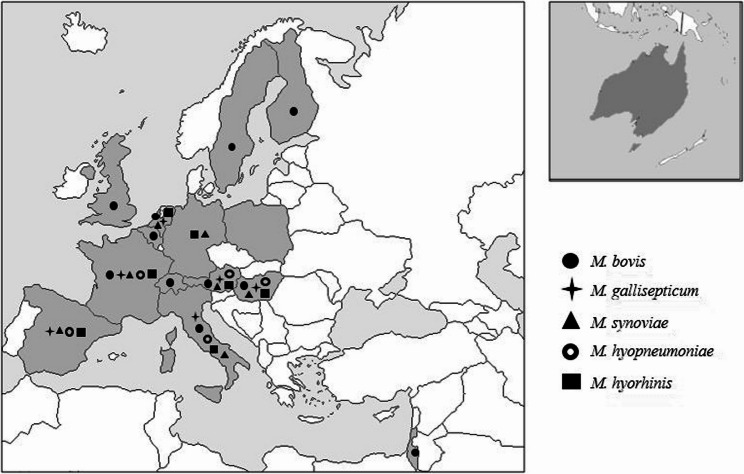
Geographical distribution of MyMIC partners that are currently conducting mycoplasma diagnosis and/or AST according to WP1 survey. Partner countries are colored in grey while those performing AST are marked with a symbol according to the *Mycoplasma* species considered

The survey was sent to all MyMIC partners, i.e. 22 institutes in 18 countries. Responses were received from 19 institutes across 15 countries -all located in Europe except for Australia- (Fig. 2). Of these, 14 institutes provided detailed insights into their practices concerning *M. bovis* (including 11 performing AST), eight on *M. synoviae* and *M. gallisepticum* (including seven and six institutes respectively performing AST) and five on *M. hyorhinis* and *M. hyopneumoniae* (all five performing AST). Some questions were unanswered or ambiguously answered and responses were therefore recorded as “not specified”.

#### *Media used for isolating Mycoplasma strains*

Description of culture media used were provided by 12/14 participants for *M. bovis*, 7/8 for *M. gallisepticum* and *M. synoviae* and 4/5 for *M. hyopneumoniae* and *Mycoplasma (M.) hyorhinis [Mesomycoplasma hyorhinis]*. Several answers mentioned the use of more than one medium for isolating a particular species (5/12 for *M. bovis*, 5/7 for *M. gallisepticum*, 4/4 for *M. synoviae*, 3/4 for *M. hyopneumoniae*, 2/4 for *M. hyorhinis*). The culture media most commonly used for the isolation of the *Mycoplasma* species included in this paper are listed in Table 1. These media include industrially produced, ready-to-use ones, as well as those prepared in-house at each institution. In-house prepared media usually included a common basal medium but their supplementation varied from one laboratory to another (Table 1). A high diversity of media used was observed among the participants, not only between *Mycoplasma* species but also for the same species. For instance, for *M. gallisepticum* up to seven different media were identified as being in use by participating laboratories.

**Table 1:** List of growth media used by the different partners for mycoplasma culture prior to AST

**Culture medium**	**Reference**	**Comments**	No. of partners using this medium per Mycoplasma species				
			***M. bovis***	***M. synoviae***	***M. gallisepticum***	***M. hyopneumoniae***	***M. hyorhinis***
APB ^®^ (registered trademark)	[33]	Patented, home-made medium by Dr Poveda laboratory	no	1	1	1	1
MolliScience General Mycoplasma media		Home-made medium	1	1	1	1	1
Modified Eaton’s medium	[34]		2	no	no	no	no
Frey and modified Frey	[20, 35]	Developed for avian mycoplasmas, specially *M. synoviae*	no	3	3	no	no
Friis and modified Friis	[36, 37]	For swine species modification consisted of supplementation by both horse serum (20%) and swine serum in identical proportions.	2	no	1	4	2
Modified Hayflick	[38]	Medium B supports the growth of most *Mycoplasma* species.	2	no	no	no	no
Mycoplasma Experience Medium (Mammal or avian)		https://www.mycoplasma-exp.com/index.html	3	4	3	2	2
PPLO and modified PPLO (includes INDICIA and Oxoid/Thermofisher agar/broth + supplement G or P as well as ready to use plate from Thermofisher)		Different commercial references are available http://www.oxoid.com/; https://indicia.fr/company/; https://www.thermofisher.com	7	1	2	no	no
SP4, SP4-Z-glucose or SP4-II	[39–41]	Various supplements for fastidious species	no	2	2	no	no
		Total	17	12	13	8	6

#### *Growth conditions for isolating Mycoplasma strains*

Conditions used for growth are summarized in Table 2. The use of a 37 °C humidified incubator with 5% CO_2_ was the most common reported practice, irrespective of laboratory and *Mycoplasma* species, for both broth and agar media. One laboratory specified incubation in this CO_2_-enriched atmosphere was preferred for agar medium only to obtain bigger colonies.

**Table 2:** Incubation conditions for *Mycoplasma* strains by species. The numbers indicate the number of laboratories in each category. All laboratories use the same incubation conditions for both broth and agar except two (reported only once in with a "*") that uses aerobic incubation for liquid medium and 37 °C humidified incubator with 5% CO2 only for culturing these bacteria on agar medium to obtain bigger colonies

	Incubation conditions				Tot no. of responding labs
	37°C			39.5°C	
	aerobic	5% CO_2_,humidified	7% CO_2;_ 7% N_2_	aerobic	
*M. bovis*	3	10 + 1*	none	none	14
*M. synoviae*	3	2 + 2*	none	1	8
*M. gallisepticum*	3	1 + 2*	1	1	8
*M. hyopneumoniae*	2	1 + 2*	none	none	5
*M. hyorhinis*	1	2 + 2*	none	none	5

The most commonly reported method for detecting growth in broth medium was the use of a pH indicator such as phenol red, employed by 11/14 institutes for *M. bovis*, all eight institutes for *M. synoviae* and *M. gallisepticum*, and all five institutes for *M. hyopneumoniae* and *M. hyorhinis*. Growth detection based on change in turbidity was less frequently reported: 3/14 institutes for *M. bovis*, 2/8 institutes for *M. synoviae*, 3/5 institutes for *M. hyopneumoniae*. Most of the partners utilized both color change and turbidity, but not systematically in combination.

#### *Methods used for *Mycoplasma* species identification*

Table 3 shows the most common methods used in routine by partners for identification of isolates. These were (i) primarily PCR-based methods, corresponding to either commercial kits or in-house methods, real-time or end-point PCRs with various subsequent analyses of the amplicons, (ii) MALDI-TOF mass spectrometry (when applicable, i.e. mainly used for easily growing species and where laboratories have access to a comprehensive *Mycoplasma* spp. database) and (iii) more rarely immune-based methods like MF-dot, immunofluorescence assay or immunobinding method (Table 3). A high diversity of genetic targets for PCR-based methods used by the partners was noted for all *Mycoplasma* species. Of note, three laboratories mentioned the use of whole genome sequencing as a complementary test for identification, of which one used nanopore sequencing directly as a diagnostic method for *M. bovis* species in general using a commercially available method (https://www.pathosense.com/).

**Table 3:** List of methods used for *Mycoplasma* species detection and/or identification

Method	Species (no. of participants detailing the method used)	Target (number of participants using the method^1^)	Reference
Molecular biology			
Endpoint or real time PCR	*M. bovis* (9)	*polC* (3), *oppD* (5), *uvrC* (3)	[42–44]
	*M. synoviae* (7)	* 16 s rRNA* (4), 16–23 S intergenic spacer region (4), *vlhA* (1)	[45–49]
	*M. gallisepticum* (6)	*Mgc2* (6), * 16 s rRNA* (1), fMG-2 (1)	[48–50]
	*M. hyopneumoniae* (3)	*I141 probe* (1), *p102* (2), 16 S rRNA (1), p97 (1)	[51–55]
	*M. hyorhinis* (2)	*p37* (2), 16 S rRNA (1)	[53, 56]
High Resolution Melting Curve analysis or Gene Targeted Sequencing	*M. synoviae* (7)	*vlhA* (2), *oppF* (1), *obg* (2), *16 S rRNA* (2), 16–23 S intergenic spacer Region (1)	[57]
16S rRNA gene PCR + Denaturing Gradient Gel Electrophoresis	All (7)	V3 region of the 16S rRNA coding gene (7)	[58–60]
Mass spectrometry			
MALDI-TOF mass spectrometry	All (12) but more difficult with poorly cultivable swine species	Protein profile	[61–64]
Immunobased methods			
Dot-immunobinding on filtration membrane	*M. bovis* (1)	Major antigens	[65]
Immunofluorescence directly on colony-bearing agar slices	*M. bovis (1)*,* M. synoviae (1)*	Major antigens	[66, 67]

^1^ Each participant may utilize multiple molecular targets for a single species

### Inventory of phenotypic AST methods applied within the mymic partners

Eleven participants for *M. bovis*, seven for *M. synoviae*, six for *M. gallisepticum*, and five for *M. hyorhinis* and *M. hyopneumoniae* declared performing phenotypical AST methods, resulting in a total of 34 answers (See supplementary Table 1 for a complete overview). The colony picking method, preferably after a second passage, was the most frequently reported method for colony selection prior to AST for *M. bovis* (7/9 answers), *M. gallisepticum* (4/6 answers), *M. synoviae* (5/6 answers), *M. hyopneumoniae* and *M. hyorhinis* (3/5 answers each).

#### Broth Dilution method (32 of 34 answers)

Broth dilution was the most frequently used AST method, with 9/11 participants for *M. bovis*, all for *M. synoviae*,* M. gallisepticum*,* M. hyopneumoniae* and *M. hyorhinis.* Preliminary titration of the inoculum was reported by most participants using broth dilution, but a variety of methods were used. Color Changing Units (CCU) determination was the most frequently reported method (four for *M. bovis*, *M. gallisepticum*, *M. hyopneumoniae*, *M. hyorhinis* and *M. synoviae*) followed by photometry - either absorbance or turbidimetric scattering measurements - (two for *M. bovis*, and one for *M. gallisepticum*, *M. hyopneumoniae*, *M. hyorhinis* and *M. synoviae*) and by CFU counts on agar plates (one for *M. bovis* and *M. synoviae*). This preliminary titration was run before and/or after storage at −70 or −80 °C of the inoculum intended for AST.

Where information was provided (*n* = 23, with six answers for *M. bovis*, four for *M. gallisepticum*, three for *M. hyopneumoniae*, four for *M. hyorhinis*, six for *M. synoviae*), the media used for broth dilution were also disparate and dependent on the species under test: PPLO (3/6 for *M. bovis*), Mycoplasma experience (2/6 for *M. bovis*, 1/4 for *M. gallisepticum*, 2/6 for *M. synoviae*, 1/3 for *M. hyopneumoniae* and 2/4 for *M. hyorhinis*), Friis medium (1/6 for *M. bovis*, 2/3 for *M. hyopneumoniae* and 2/4 *M. hyorhinis*), modified Frey medium (2/4 for *M. gallisepticum*, 2/6 for *M. synoviae)*, SP4 (1/6 for *M. synoviae*), APB (1/4 for *M. hyorhinis*, 1/4 for *M. gallisepticum* and 1/6 for *M. synoviae*). Commercial MIC plates, preloaded with dried standardized antimicrobial dilutions, were used among participants especially for *M. bovis* (*n* = 8), *M. gallisepticum* (*n* = 4) and *M. synoviae* (*n* = 5), *M. hyopneumoniae* (*n* = 2) and *M. hyorhinis* (*n* = 3), but in-house plates, where antimicrobial dilutions were prepared in-house just before use, were favored for *M. hyopneumoniae* (*n* = 3) and *M. hyorhinis* (*n* = 2). Incubation in aerobic conditions was preferred to CO_2_ enriched conditions - that might result in acidification of the broth and color change nonrelated to growth-, with the exception of four participants for *M. bovis*.

While performing AST, a growth control well (without any antimicrobial) was included (all 25 responses), while a negative control (sterile medium) was not systematically included (17/25 answers). The bacterial load after the assay was most commonly validated by determining CCU (*n* = 16) or, alternatively, by colony counts on agar plates (*n* = 3). Some laboratories also used photometry (*n* = 5), whereas the remainder do not control the inoculum in each run (*n* = 8).

Plates were checked daily for most participants and rarely twice a day (one for *M. hyopneumoniae*, *M. hyorhinis*, *M. gallisepticum* and *M. synoviae*). The final reading was performed according to the growth of the control well and within a limited period which ranged from 24 to 72 h – with the exception of one laboratory for which the incubation was run for one week- for *M. bovis* and *M. synoviae*, two to five days for *M. gallisepticum*,* M. synoviae* and *M. hyorhinis* and three to 14 days for *M. hyopneumoniae*. Growth was mostly assessed by detection of color change (19/19 answers to this question), detected by naked eye, with, or without, an inverted mirror or light. The detection of turbidity by visual observation was additionally mentioned by four participants. Each strain was usually tested only once (13 out of 32 answers) or two (*n* = 11) to three times (8 out of 32 answers).

The EUCAST-recommended full range of concentrations for broth dilution methods were tested less frequently than truncated ranges (14 versus 19 answers, respectively). This related to the use of commercial plates which - even when customized- are optimized for a maximum number of antimicrobials to be tested rather than wide concentration ranges. Of 22 answers reporting the use of commercial plates, 13 reported a truncated range - i.e. a MIC distribution with MIC values given as ≤ x mg/L at the lower end of the tested range or as > y mg/L at the upper range of the tested range.

#### Agar Dilution method (three of 34 answers)

Agar dilution methodology was implemented in only three laboratories (two for *M. bovis* and one for *M. hyorhinis*). Preliminary titration, as a means of standardizing the inoculum of tested strains was mentioned by two out of the three laboratories. Colony counts should fall within 30–300 CFU per spot [18]. The agar media used were PPLO for *M. bovis* (*n* = 1) or Hayflick (*n* = 1) and Friis (*n* = 1) for *M. hyorhinis.* All participants incubated the agar plates in a 5% CO_2_ enriched atmosphere. Each strain was tested two to three times and plates were read after 4–5 days of incubation at 37 °C for *M. bovis* and 72 h at 35–37 °C for *M. hyorhinis*. Full range of concentrations according to EUCAST recommendations - i.e. from 0.03 to 256 mg/L- were tested by the three participants using this method as well as a truncated range (*n* = 1).

Irrespective of the AST method, i.e. broth versus agar dilution, the use of a control strain, with known MICs, was reported in 23/27 responses (seven for *M. bovis*, four for *M. gallisepticum* and five for *M. synoviae*, three for *M. hyopneumoniae* and *M. hyorhinis*). Control strains mostly belong to the tested species with a majority reporting the use of reference, as opposed to field strains (*n* = 17/23). However some respondents reported using controls belonging to another species (for instance *M. gallisepticum* was reported to be used as a control for *M. hyorhinis*, *M. hyopneumoniae* and *M. synoviae*) or genus (for instance *Escherichia coli*, *Enterococcus faecalis*, and *Staphylococcus aureus*).

### First evaluation of raw MIC datasets provided by MyMIC partners for their potential to support tentative ECOFFs definition

MIC distributions intended to be imported into the EUCAST database for aggregation purpose as well as the underlying methodology used to obtain the data must fulfil a number of basic requirements [19]. Most laboratories of the MyMIC consortium used either broth or agar microdilution approaches based on Hannan, 2000 [16]. However, methodologies differed in respect to the media used, either commercial (Mycoplasma Experience, Indicia or Oxoid) or in-house prepared (Frey, Friis, modified PPLO broth, etc.), with or without supplements. Other important variables included the incubation time, the control strains used and the antimicrobials as well as their concentration ranges tested. Table 4 illustrates the variety of methods used for *M. bovis* AST alone across MyMIC partners. Some in-house prepared media have similar names (Frey, Frey medium 4) as they refer to the same original publication [20] and therefore, it will be necessary to check the exact composition of each medium to determine the exact formulation and assess comparability.

**Table 4:** Summary of antimicrobial susceptibility testing methods applied to *M. bovis *by MyMIC participating partners across multiple antimicrobial classes

				Antibiotic classes tested									
Lab	Method & Media	Incubation time (h)	Control strains	Aminocyclitols^1^	Amino-glycosides^2^	Fluoro-quinolones^3^	Lincosamides^4^	Macrolides 14-membered rings^5^	Macrolides 15-membered rings^6^	Macrolides 16-membered rings^7^	Phenicols^8^	Pleuromutilins^9^	Tetracyclines^10^
Lab 1	Agar dilution Indicia	72 h	*M. bovis* ATCC 25,223 *M. gallisepticum* ATCC 15,302	X		X			X	X	X		X
Lab 2	Broth dilution MB	24–120 h	*M. bovis* ATCC 25,223 Internal strains			X				X			
Lab 3	Broth dilution modified PPLO broth	48–72 h	*M. bovis* ATCC 25,223 *S. aureus* ATCC 29,213 *E. coli* ATCC 25,922		X				X	X	X	X	X
Lab 4	Broth dilution Avian mycoplasma experience without inhibitors	24–72 h	*M. bovis* ATCC 25,223	X		X	X	X		X	X	X	X
Lab 5	Broth dilution Friis (without antibiotics)	48 h	*M. bovis* ATCC 27,368			X	X		X	X			X
Lab 6	Broth dilution Oxoid	24–168 h	*M. bovis* ATCC 25,223	X	X	X	X		X	X	X	X	X
Lab 7	Broth dilution Mycoplasma broth with Mycoplasma select. supplement-G Oxoid	72 h	*M. bovis* ATCC 25,223			X	X	X	X		X		X
Lab 8	Broth dilution *M. bovis* media	168–240 h	*M. bovis* ATCC 25,223			X		X	X	X	X		X
Lab 9	Broth dilution *M. bovis* media	48 h	*M. bovis* ATCC 25,223			X		X	X	X	X		X

^1^ spectinomycin

^2^ gentamicin

^3^ danofloxacin, enrofloxacin, levofloxacin, marbofloxacin, pradofloxacin

^4^ clindamycin, lincomycin, pirlimycin

^5^ clarithromycin, erythromycin

^6^ azithromycin, garnithromycin, tulathromycin

^7^ spiramycin, tildipirosin, tilmicosin, tylosin, tylvalosin

^8^ florfenicol

^9^ tiamulin, valnemulin

^10^ doxycycline, oxytetracycline, tetracycline

Most laboratories that provided raw MIC data mentioned the use of control strains (18/29 answers), which were, in most cases, identical within each species, even if different names were provided (for example *M. synoviae* ATCC 25204 = NCTC 10124 = WVU 1853), *M. hyopneumoniae* ATCC 25934 = NCTC 10110 = J type strain or *M. bovis* ATCC 25523 = PG45 type strain). MIC values obtained for several antimicrobials on control strains were mainly grouped within a range of 2–3 dilutions (Table 5), despite the fact that different media (commercial or in-house) and different techniques (agar or broth dilution) were used (see for example results obtained for enrofloxacin, tylosin and florfenicol with strains of *M. bovis*). In contrast, very different results were observed for some antimicrobial-*Mycoplasma* species combinations: e.g. oxytetracycline and *M. bovis* or enrofloxacin and *M. synoviae* (Table 5).

**Table 5:** MIC values (in mg/L) for four antimicrobials, obtained from anonymised laboratories participating to MyMIC, for the quality control strains listed below. MS, *M. synoviae* ATCC 25204; MHp, *M. hyopneumoniae* ATCC 25934; MB, *M. bovis* ATCC 25523 ND, not determined or not provided All MIC values were obtained with a broth microdilution method, except values from lab. 1 which used an agar dilution method for *M. bovis*. Different media were used for the broth dilutions: for example, avian Mycoplasma Experience (lab. 4), FM4 (lab. 1), Frey (lab. 2 and 6) for MS; Friis (lab. 1) or Mycoplasma Experience (lab. 6) for MHp; Mycoplasma Experience (lab. 2 and 8) or Oxoid (lab. 6) for MB

	Enrofloxacin			Tylosin			Oxytetracycline			Florfenicol		
	MS	MHp	MB	MS	MHp	MB	MS	MHp	MB	MS	MHp	MB
Lab. 4	2	ND^c^	ND	0.0156	ND	ND	< 0.5	ND	ND	1	ND	ND
Lab. 1	0.5	≤ 0.03	0.25	≤ 0.03	0.125	1	0.06	0.125	2	1	0.25	2
Lab. 6	0.312	≤ 0.039	0.156	≤ 0.25	≤ 0.25	0.5	≤ 0.25	≤ 0.25	2	1–2	1	4
Lab. 2	0.5	ND	0.16	0.03	ND	ND	ND	ND	ND	ND	ND	ND
Lab. 10	ND	ND	ND	ND	0.06	ND	ND	ND	ND	ND	ND	ND
Lab. 7	ND	ND	0.25	ND	ND	ND	ND	ND	ND	ND	ND	4–8
Lab. 8	ND	ND	0.25	ND	ND	ND	ND	ND	32	ND	ND	8
Lab. 3	ND	ND	ND	ND	ND	ND	ND	ND	ND	ND	ND	ND

**Fig. 3:**
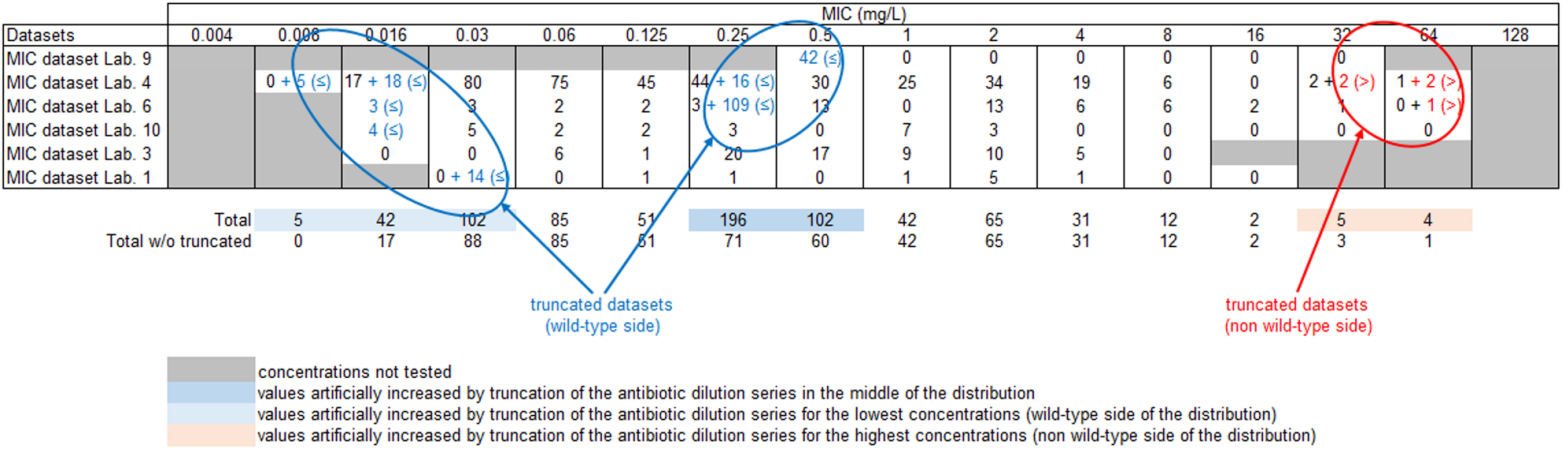
Example of MIC distributions of datasets provided by six MyMIC-participating laboratories for tylosin/M. synoviae without preliminary harmonization of the AST methodology

Within the MIC results provided by the MyMIC partners for field strains, some antimicrobial-mycoplasma species combinations had many data, while other combinations were tested by few, or even a single laboratory, impeding any attempts to aggregate distribution data. The MIC distribution patterns for the different field isolates were influenced by the variations in the AST methods (Fig. 3). For instance, in addition to the different media and control strains used (if any), the laboratories did not always use the same concentration range or incubation time and truncated distributions prevented the aggregation of data.

## Discussion

In the overall diagnostics workflow for bacterial diseases, AST of the main identified causative agent is an essential step so clinicians can set more effective treatments, i.e. treatments with a higher chance of clinical success considering the level of susceptibility of specific pathogens to a wide range of antimicrobial agents [21]. To serve this purpose not only standardized laboratory methods and robust interpretative criteria are required, but also effective and consistent communication between microbiologists and field veterinarians.

The survey of veterinary practices revealed several concerns regarding best practices for mycoplasma infections, as treatments without prior diagnosis -including AST- remain relatively common, though varying by livestock sector.

The main antimicrobial classes used when mycoplasma infections are suspected include macrolides, tetracyclines, phenicols, pleuromutilins, regardless of the livestock sector, while fluoroquinolones are typically used following treatment failure in cattle. These choices primarily reflect the framework of marketing authorizations for antimicrobial agents. Such authorizations typically consider the extensive intrinsic resistances of mycoplasmas [11] but insufficiently address their ability to acquire additional resistance mechanisms. For example, in the case of *M. bovis*, markedly elevated MIC values have been reported for several antimicrobial classes in several countries [10, 11], including macrolides. The continued use of macrolides may be driven more by their anti-inflammatory properties than by their antimicrobial efficacy [22–24]. Although fluoroquinolones have generally retained low MICs against *M. bovis* and may represent a potential therapeutic option, their use is restricted due to their classification as critically important for human medicine and should only be considered when no alternative treatments are available [14]. In the absence of CBPs, the rationale for selecting appropriate treatments should more thoroughly incorporate antimicrobial resistance surveillance data specific for each livestock sector and country, with ECOFFs—when available— offering additional guidance for practitioners. A no treatment option – in case of moderate clinical signs- was also mentioned by several veterinarians, especially for poultry and swine, which should also be considered with caution in light of animal welfare concerns.

A clear discrepancy can be noted in between 54 and 72% veterinarians, depending on the livestock sector, requesting AST from time to time versus a mean of 33% veterinarians (all sectors combined) requesting it often or always, suggesting that the latest have an easy access to laboratories being able to actually run AST. The relative paucity of laboratories able to routinely perform AST might also be a hurdle to more frequent AST requests by veterinarians, in addition to the lack of CBPs. The establishment of routine-compatible diagnostic tests in all sectors as well as guidelines for a standard, reference method for AST will certainly help laboratories to set up AST, as already emphasized for other bacteria of veterinary importance [22]. However, gold standard AST methods are often time-consuming and hence incompatible with the necessity to take everyday treatment decisions, particularly considering the need for an early treatment in the case of mycoplasmosis. Furthermore, the use of PCR as preferred technique for mycoplasma detection in first-line diagnostic laboratories, as revealed by the survey here, is a major hindrance to phenotypic AST, which requires isolation of strains. To ensure broad accessibility of mycoplasma AST, method validation must consider the practical and economic constraints faced by frontline laboratories. Establishing ECOFFs could enable the design of standardized AST plate formats tailored to each mycoplasma species, with antimicrobial dilution ranges optimized around the ECOFFs, thus supporting the development of ready-to-use commercial testing plates.

The survey on diagnostic procedures within specialized laboratories – i.e. MyMIC partners in contrast to first-line laboratories – revealed a high diversity of methods used for culturing and identifying mycoplasma isolates. This diversity is due to the different nutritional requirements of the various species [1] but is also related to the individual history of each laboratory regarding their mycoplasmology know-how, resulting in multiple, apparently minor variations in recipes to prepare growth media. While media variability has limited impact on cultivation prior to identification, it can compromise the comparability of MIC values in AST. Mycoplasma media typically contain animal-derived complex components -such as brain heart infusion, beef extracts, peptones, pancreatic digest of casein, and serum-, which can also affect batch-to-batch reproducibility. Chemically defined, serum-free media are usually developed only for vaccine production [25]. In addition to growth conditions, substantial variability among laboratories was observed in inoculum density, incubation time, antimicrobial dilution ranges, and use of quality control strains, all of which can compromise the consistency of MIC results across laboratories. A comparative study of different AST methods (e.g., broth versus agar dilution) using well-characterized strains could help guide recommendations to reduce inter-laboratory variability and promote best practices.

MIC data provided by MyMIC partner laboratories showed some deviations, primarily due to the use of different media and varying incubation times. For instance, in the case of oxytetracycline, which is a bacteriostatic antimicrobial known to degrade rapidly, the different incubation times used (Table 4), might be the cause of the important differences observed between results of laboratories for the control strain of *M. bovis* with a higher MIC value 32 mg/mL (lab. 8) versus 2 mg/mL (lab. 1 and 6) when the reading time is higher (Table 5). The use of shortened concentration ranges prevented us from determining tentative ECOFFs for all antibiotic-mycoplasma combinations. However, analysis, in collaboration with the EUCAST steering committee, is ongoing on a promising subset of MIC data, acceptable for creating distributions and defining tentative ECOFFs. Together with the current results, this analysis will be valuable for developing AST guidelines that recommend appropriate media and/or media suppliers –amongst the ones used by MyMIC partners-, optimal reading times and the establishment of control ranges for selected control strains for each *Mycoplasma* species. These guidelines will be further reinforced through two-laboratory and subsequently five-laboratory ring trial studies.

Because gold standard AST methods rely on in-house antimicrobial stock solutions and involve extensive preparation, frontline laboratories are often inclined to use ready-to-use commercial plates instead [26]. However, such commercial systems often fail to include the complete range of antimicrobial concentrations or all molecules of clinical relevance. Including the most-often-used molecules in AST routine is of importance to assist veterinarians in decision making and for monitoring resistance trends over time. Currently, most of the standard “off-the-shelf” commercial plates for AST in veterinary medicine target either Gram-positive or Gram-negative bacteria, but not mycoplasmas. Consequently, they do not include pleuromutilins –demonstrated to be used regularly in the swine and poultry sectors- but comprise other antimicrobials with little relevance to *Mycoplasma* spp. Moreover, ordering customized plates remains expensive today and limited to laboratories with a sufficient number of analyses. Besides, reading microbroth dilution commercial plates for mycoplasmas requires some training especially for species showing weak color change/turbidity. Therefore, training first-line laboratories must be an essential part of the roadmap for improving mycoplasma AST.

Whether genotyping –in contrast to phenotypic- AST could be considered a valuable solution in routine diagnostic laboratories is also debated. MIC data are increasingly being supported by molecular evidence of genetic mutations consistent with antimicrobial resistance, thus many molecular methods targeting these mutations have been proposed lately, all claiming to be robust, rapid and cost-effective. This topic is currently being addressed in another article from the MyMIC consortium (Sulyok et al., in preparation).

Interpretation of phenotypic AST results relies on CBPs, where ECOFFs are part of the basis for the definition of CBPs [27]. An additional objective initially proposed in the framework of MyMIC was to gather PK/PD data for the determination of CBPs used as interpretation criteria. However, few time-kill curves, which provide essential data needed for determination of CBPs, were readily available in partners’ laboratories and there is general paucity of essential PK/PD data. Obtaining this additional information would have required extensive laboratory work, which was beyond the scope of this project, so this objective was abandoned and MyMIC focused on ECOFFs only. At the time of writing, only a few distributions resulting in tentative ECOFF values were available on the EUCAST site for *Mycoplasma* species of animal origin (https://mic.eucast.org/search): one for *M. hyopneumoniae*/florfenicol and two for *M. bovis*/florfenicol and *M. bovis*/enrofloxacin. By comparison, more than 15 years ago the CLSI mandated 10 laboratories for establishing AST guidelines for three human mycoplasma species, i.e. the cultivable *M. hominis*, *M. pneumoniae* and *U. urealyticum*, which resulted in the publication of the CLSI reference broth dilution method (M43-A for Human mycoplasmas) [17]. At that time, even with only three species, and because of the growth medium complexity, some derogations were requested from the stringent requirements of the CLSI and the authors clearly stated that procedures and references should be limited to testing only the species for which they are described [17]. This makes the MyMIC challenge even greater considering the large number of pathogenic *Mycoplasma* species in livestock, each requiring its own standard for growth, as illustrated here. Here, only five major species were considered -whose relevance stems from their global distribution in livestock- but there are other species posing significant health and economic challenges that should be considered for antimicrobial resistance over the more than 140 *Mycoplasma* species discovered to date [1]. This refers primarily to major pathogens with a limited geographic distribution, such as *M. mycoides* subspecies *mycoides*, the agent of Contagious Bovine Pleuropneumonia (CBPP), or *M. capricolum* subsp. *capripneumoniae*, the agent of Contagious Caprine Pleuropneumonia (CCPP), and to the four (sub)species involved in contagious agalactia of small ruminants, namely *M. agalactiae* [*Mycoplasmopsis agalactiae*], *M. capricolum* subsp. *capricolum*, *M. mycoides* subsp. *capri* and *M. putrefaciens*. CPBB, CCPP and contagious agalactia are WOAH-listed diseases [28]. In addition to these highly pathogenic mycoplasmas, other less virulent mycoplasmas, which are widely distributed, contribute to chronic clinical conditions that adversely affect animal welfare and result in economic losses. Some examples are *M. dispar* [*Mesomycoplasma dispar*] [29], *M. ovipneumoniae* [*Mesomycoplasma ovipneumoniae*] [30] and *M. hyosynoviae* [*Metamycoplasma hyosynoviae*] [31]. Consequently, this study should be viewed as the initial stage of a long and necessary process.

## Conclusions

In conclusion, the MyMIC project has, to date, identified and analyzed some important gaps between what laboratories -either expert or first-line- can currently offer in terms of diagnosis and AST for livestock mycoplasmas and the requirements of veterinarians to determine the most effective treatments. The consortium of MyMIC expert laboratories counts now with a roadmap listing several necessary steps required to be able to produce both methodological guidelines for implementating standardized AST as well as to establish ECOFFs to inform veterinarians on the most appropriate treatment. Ring trials based on consensus methodologies developped from the partners’ expertise for each *Mycoplasma* species, as well as the design of ready-to-use commercial plates, will be the next steps.

## Materials and methods

### Survey on field practices regarding diagnosis and antimicrobial use for controlling mycoplasmoses

A survey was designed using Formdesk^®^ (https://formdesk.com/en/) to collect data on antimicrobials that are used by veterinarians worldwide for the treatment of mycoplasmas’ infections in different livestock sectors, i.e. bovine, swine and poultry. It consisted of 23 questions that aimed at gathering data on the veterinarians’ profiles, their practices regarding mycoplasma diagnosis, their preferences for the treatment (first choice versus in case of failure) and the rationale for these choices (see supplementary data 1). The survey was made available online between April 22nd and September 30th 2024, via a QR code or weblink in different languages (Dutch, English, Finnish, French, German, Hungarian, Italian, Polish, Portuguese, Spanish and Swedish). The survey was advertised to veterinary associations worldwide for distribution to their members, both generalists and specialists in different livestock sectors, and to veterinary diagnostic laboratories. Other contacts were made through the networks of MyMIC members. The survey was mainly distributed by e-mail and newsletters, but also during a few national and international congresses such as the IOM (International Organization for Mycoplasmology) congress and AAVM (Antimicrobial Agents in Veterinary Medicine), and through professional magazines. After the questionnaire was closed, the data were exported from Formdesk^®^ into a Microsoft^®^ Excel file and then separated into individual files for each animal species. Percentage calculations and graphical analyses were performed using R [32]. In this work, only a partial analysis of the answers to four questions was conducted, focusing on (i) an inventory of antimicrobial agents prescribed with regard to mycoplasma infections, (ii) whether these infections were confirmed by laboratory diagnosis before treatment and (iii) whether AST was performed regularly. A distinction was made between animal species (poultry, swine and ruminants) and conditions of use (first-line treatment versus treatment after a first treatment failure). The detailed analysis of the complete survey results per animal species will be published separately.

### Survey of the current practices in culture, identification and AST in partners’ laboratories

Another comprehensive online survey was distributed to MyMIC network partners to document their diagnostic practices for *M. bovis*, *M. synoviae*, *M. gallisepticum*, *M. hyorhinis* and *M. hyopneumoniae* as well as potential additional species of interest (not presented here). The survey was sent to MyMIC partner contact points (one to three per institute) with the instructions to provide one answer per institute and per mycoplasma species. It aimed to collect information on methodologies used in different laboratories for culture, identification, and AST of animal mycoplasmas. More specifically, for culture, participants were asked to detail media type, form (agar or broth) and origin (commercial or in-house), incubation conditions as well as growth indicators. Regarding identification, participants were surveyed about the methods used by providing information on principle, target, preliminary steps (culture, extraction, etc.) and controls used. For AST, both phenotypical and molecular characterization were explored. For phenotypic AST, strain preparation and calibration, media used, antimicrobial range (full or truncated), growth and reading conditions, controls, validation criteria, and interpretation were investigated. For molecular and genomic approaches, partners had to specify method, preliminary steps, controls and interpretation. The survey design allowed to include several answers for questions about methods to assess the diversity of practices within each laboratory.

Each survey response was identified by the institute name, mycoplasma species, and provider. The results were checked for duplicates accordingly (one response expected per institute and mycoplasma species) and analysis was performed using Excel.

### MIC data gathering, quality control and aggregation

The preceding survey allowed the identification of MyMIC partners willing to share available raw MIC data generated using methods described in detail, including the publication and intellectual property status of these data. An Excel file was prepared and distributed among each of these partners to be filled in. Information to be provided in the file included: *Mycoplasma* species tested, details on the AST methodology, such as culture medium used, incubation atmosphere and time before MIC reading, and a list of *Mycoplasma* isolates along with the MIC values of all antimicrobials tested, including internal control strains and their MIC profile. In addition, the dilution range tested for each of the antimicrobials was also requested. MIC data from the partners were anonymized and analyzed in detail looking closely at the different experimental steps that could influence the results. Where possible (comparable methods, MIC value for quality control strains, etc.), harmonized MIC data from different laboratories were aggregated. A first evaluation was conducted on these overall data to clarify the necessary improvement needed to reach distribution compliant for ECOFF setting according to the criteria of EUCAST [19].

## Supplementary Information

 Additional file 1.pdf: Text of the survey on field practices regarding diagnosis and antimicrobial use for controlling mycoplasmoses as sent out to veterinarians

Additional file 2.pdf: Text of the survey of the current practices in terms of culture, identification and AST as filled in by partners’ laboratories

Additional Table 1: Detailed methodologies used in MyMIC laboratories to perform antimicrobial susceptibility testing per *Mycoplasma* species.

## Data Availability

The authors confirm that all supporting data and protocols have been provided within the article or through supplementary data files. Details on surveys results are available upon request to the corresponding author.

## Abbreviations

AST: Antimicrobial susceptibility testing
CBPs: Clinical breakpoints
CBPP: Contagious Bovine Pleuropneumonia
CCPP: Contagious Caprine Pleuropneumonia
CLSI: Clinical & Laboratory Standards Institute
ECOFF: Epidemiological cut-off
EUCAST: European committe on antimicrobial suscpetibility testing
M.: Mycoplasma
MIC: Minimun inhibitory concentration
PK/PD: Pharmacokinetic/pharmacodynamic
WOAH: World Organisation for Animal Health

## References

[1] Balish MF, Chopra-Dewasthaly R, Pereyre S, Ramirez AS, Viver T, Spergser J. Bergey’s manual of systematics of archaea and Bacteria, online [Internet]. John Wiley & Sons, Inc., in association with Bergey’s Manual Trust; 2024. 10.1002/9781118960608.gbm01263.pub3. Mycoplasma.

[2] List of Prokaryotic names with Standing in Nomenclature (LPSN) moves to the DSMZ [Internet]. 2020 [cited July 21 2025]. 10.1099/ijsem.0.004332 PMC7723251 32701423

[3] Yan XH, Pei SC, Yen HC, Blanchard A, Sirand-Pugnet P, Baby V et al. Delineating bacterial genera based on gene content analysis: a case study of the Mycoplasmatales-Entomoplasmatales clade within the class mollicutes. Microb Genom. 2024;10(11):001321. 10.1099/mgen.0.001321. 10.1099/mgen.0.001321 PMC11567158 39546405

[4] Freundt E. The classification of the pleuropneumonia group of organisms (Borrelomycetales). Int Bull Bacteriological Nomenclature Taxonomy. 1955;5:67–8.

[5] Nowak J. Morphologie, nature et cycle évolutif du microbe de La péripneumonie des bovidés. Ann De l’Institut Pasteur (Paris). 1929;43:1330–52.

[6] Gupta RS, Sawnani S, Adeolu M, Alnajar S, Oren A. Correction to: Phylogenetic framework for the phylum tenericutes based on genome sequence data: proposal for the creation of a new order Mycoplasmoidales ord. nov., containing two new families Mycoplasmoidaceae fam. nov. And Metamycoplasmataceae fam. nov. Harbouring Eperythrozoon, Ureaplasma And five novel genera. Antonie Van Leeuwenhoek. 2018;111(12):2485–6. 30328003 10.1007/s10482-018-1175-9

[7] Hoelzle K, Ade J, Hoelzle LE. Persistence in livestock Mycoplasmas—A key role in infection and pathogenesis. Curr Clin Microbiol Rep. 2020;7:81–9.

[8] Feberwee A, von Banniseht-Wysmuller T, Vernooij JC, Gielkens AL, Stegeman JA. The effect of vaccination with a bacterin on the horizontal transmission of *Mycoplasma gallisepticum*. Avian Pathol. 2006;35(1):35–7. 16448940 10.1080/03079450500465700

[9] Pieters MFE, Pijoan C, Dee S. An experimental model to evaluate *Mycoplasma hyopneumoniae* transmission from asymptomatic carriers to unvaccinated and vaccinated Sentinel pigs. Can J Vet Res. 2010;74:157–60. 20592848 PMC2851728

[10] Gautier-Bouchardon AV. Antimicrobial resistance in *Mycoplasma* spp. Microbiol Spectr. 2018;6(4). 10.1128/microbiolspec.ARBA-0030-2018. 10.1128/microbiolspec.arba-0030-2018 PMC11633602 30003864

[11] Pereyre S, Tardy F. Integrating the human and animal sides of Mycoplasmas resistance to antimicrobials. Antibiot (Basel). 2021;10(10):1216. 10.3390/antibiotics10101216. 10.3390/antibiotics10101216 PMC8532757 34680797

[12] Jay M, Poumarat F, Colin A, Tricot A, Tardy F. Addressing the antimicrobial resistance of ruminant Mycoplasmas using a clinical surveillance network. Front Vet Sci. 2021;8:667175. 34195247 10.3389/fvets.2021.667175 PMC8236625

[13] Dudek K, Bednarek D, Ayling RD, Kycko A, Reichert M. Preliminary study on the effects of enrofloxacin, flunixin Meglumine and pegbovigrastim on *Mycoplasma Bovis* pneumonia. BMC Vet Res. 2019;15(1):371. 31655595 10.1186/s12917-019-2122-3 PMC6815429

[14] Commission notice: Guidelines for the prudent use of antimicrobials in veterinary medicine. (2015).

[15] Koeth LM, Miller LA. Antimicrobial Susceptibility Test Methods :Dilution and Disk Diffusion Methods. In: Press A,Manual of Clinical Microbiology, 13th Edition, Washington. DC.2023. p. pp. 12 – 5. 10.1002/9781683670438.mcm0074.

[16] Hannan PCT. Guidelines and recommendations for antimicrobial minimum inhibitory concentration (MIC) testing against veterinary Mycoplasma species. Vet Res. 2000;31(4):373–95. 10958240 10.1051/vetres:2000100

[17] Waites KB, Duffy LB, Bebear CM, Matlow A, Talkington DF, Kenny GE, et al. Standardized methods and quality control limits for agar and broth microdilution susceptibility testing of *Mycoplasma pneumoniae*, *Mycoplasma hominis*, and *Ureaplasma urealyticum*. J Clin Microbiol. 2012;50(11):3542–7. 22915608 10.1128/JCM.01439-12 PMC3486213

[18] Clinical and Laboratory Standards Institute (CLSI) 2011 Methods for Antimicrobial Susceptibility Testing for Human Mycoplasmas; Approved Guideline M43-A (internet). Clinical and Laboratory Standards Institute, Wayne PA, editor. CLSI standards : Guidelines for Health Care Excellence. 31339681

[19] European Committee on Antimicrobial Susceptibility Testing (EUCAST). MIC distributions and the setting of epidemiological cut off (ECOFF) values. [Internet]. 2021.

[20] Frey ML, Hanson RP, Andrson DP. A medium for the isolation of avian Mycoplasmas. Am J Vet Res. 1968;29(11):2163–71. 5693465

[21] Wenzler E, Maximos M, Asempa TE, Biehle L, Schuetz AN, Hirsch EB. Antimicrobial susceptibility testing: an updated primer for clinicians in the era of antimicrobial resistance: insights from the society of infectious diseases pharmacists. Pharmacotherapy. 2023;43(4):264–78. 36825480 10.1002/phar.2781

[22] Bartram DJ, Moyaert H, Vanimisetti BH, Ramage CP, Reddick D, Stegemann MR. Comparative efficacy of tulathromycin and Tildipirosin for the treatment of experimental *Mycoplasma Bovis* infection in calves. Vet Med Sci. 2016;2(3):170–8. 29067192 10.1002/vms3.31 PMC5645867

[23] Catania S, Bilato D, Gobbo F, Granato A, Terregino C, Iob L, et al. Treatment of eggshell abnormalities and reduced egg production caused by *Mycoplasma synoviae* infection. Avian Dis. 2010;54(2):961–4. 20608549 10.1637/9121-110309-Case.1

[24] Kempf I, ReeveJohnson L, Gesbert F, Guitter M. Efficacy of Tilmicosin in the control of experimental *Mycoplasma gallisepticum* infection in chickens. Avian Dis. 1997;41(4):802–7. 9454912

[25] Burgos R, Garcia-Ramallo E, Shaw D, Lluch-Senar M, Serrano L. Development of a Serum-Free medium to aid Large-Scale production of Mycoplasma-Based therapies. Microbiol Spectr. 2023;11(3):e0485922. 37097155 10.1128/spectrum.04859-22 PMC10269708

[26] Pereyre S, Henin N, Dolzy A, Guiraud J, Laurier-Nadalie C, Gardette M, et al. Evaluation of commercial, customized microdilution plates for Ureaplasma parvum, Ureaplasma urealyticum, and Mycoplasma hominis antimicrobial susceptibility testing and determination of antimicrobial resistance prevalence in France. J Clin Microbiol. 2024;62(7):e0022624. 38832769 10.1128/jcm.00226-24 PMC11324033

[27] Toutain PL, Bousquet-Melou A, Damborg P, Ferran AA, Mevius D, Pelligand L, et al. En route towards European clinical breakpoints for veterinary antimicrobial susceptibility testing: A position paper explaining the VetCAST approach. Front Microbiol. 2017;8:2344. 29326661 10.3389/fmicb.2017.02344 PMC5736858

[28] WOAH. Animal diseases. 2024. https://www.woah.org/en/what-we-do/animal-health-and-welfare/animal-diseases/

[29] Dudek K, Nicholas RAJ. Recent role of microorganisms of the mollicutes class in the etiology of bovine respiratory disease. Pathogens. 2024;13(11):951. 39599504 10.3390/pathogens13110951 PMC11597336

[30] Maksimovic Z, Rifatbegovic M, Loria GR, Nicholas RAJ. *Mycoplasma ovipneumoniae*: A most variable pathogen. Pathogens. 2022;11(12). 10.3390/pathogens11121477 PMC9781387 36558811

[31] Bunger M, Blumlinger M, Loncaric I, Rosel AC, Ruppitsch W, Teich K, et al. Multilocus sequence typing schemes for the emerging swine pathogen *Mycoplasma hyosynoviae*. Vet Microbiol. 2024;290:109997. 38237446 10.1016/j.vetmic.2024.109997

[32] Core Team R, Austria V. R: A Language and environment for statistical computing. Vienna, Austria: R Foundation for Statistical Computing; 2023. https://www.R-project.org/.

[33] Poveda JB. inventorAPB MEDIUM2019.

[34] Nicholas R, Baker S. Recovery of Mycoplasmas from animals. Methods Mol Biol. 1998;104:37–43. 9711638 10.1385/0-89603-525-5:37

[35] Kreizinger Z, Grozner D, Sulyok KM, Nilsson K, Hrivnak V, Bencina D, et al. Antibiotic susceptibility profiles of *Mycoplasma synoviae* strains originating from central and Eastern Europe. BMC Vet Res. 2017;13(1):342. 29149886 10.1186/s12917-017-1266-2 PMC5693497

[36] Friis NF. Some recommendations concerning primary isolation of *Mycoplasma suipneumoniae* and *Mycoplasma flocculare* a survey. Nord Vet Med. 1975;27(6):337–9. 1098011

[37] Assuncao P, De la Fe C, Kokotovic B, Gonzalez O, Poveda JB. The occurrence of Mycoplasmas in the lungs of swine in Gran Canaria (Spain). Vet Res Commun. 2005;29(6):453–62. 16215836 10.1007/s11259-005-1866-3

[38] Hayflick L. Tissue cultures and Mycoplasmas. Tex Rep Biol Med. 1965;23(Suppl 1):285. 5833547

[39] Bradbury JM. Recovery of Mycoplasmas from birds. Methods Mol Biol. 1998;104:45–51. 9711639 10.1385/0-89603-525-5:45

[40] DSMZ. SP4-Z culture media receipe https://bacmedia.dsmz.de/medium/1076b2025 [.

[41] Ramírez AS, González M, Déniz S, Fernández A, Poveda JB. 1997 Evaluation of a modified SP-4 medium in the replication of Mycoplasma spp. In: J. Frey and K. Sarris (eds.), Mycoplasmas of Ruminants: Pathogenicity, Diagnostics, Epidemiology and Molecular Genetics, vol. 2, (European Cooperation on Scientific and Technical Research, Luxembourg), 36–39.

[42] Marenda MS, Sagne E, Poumarat F, Citti C. Suppression subtractive hybridization as a basis to assess *Mycoplasma agalactiae* and *Mycoplasma Bovis* genomic diversity and species-specific sequences. Microbiology. 2005;151(Pt 2):475–89. 15699197 10.1099/mic.0.27590-0

[43] Subramaniam S, Bergonier D, Poumarat F, Capaul S, Schlatter Y, Nicolet J, et al. Species identification of *Mycoplasma Bovis* and *Mycoplasma agalactiae* based on the UvrC genes by PCR. Mol Cell Probes. 1998;12:161–9. 9664578 10.1006/mcpr.1998.0160

[44] Sachse K, Salam HS, Diller R, Schubert E, Hoffmann B, Hotzel H. Use of a novel real-time PCR technique to monitor and quantitate *Mycoplasma Bovis* infection in cattle herds with mastitis and respiratory disease. Vet J. 2010;186(3):299–303. 19926318 10.1016/j.tvjl.2009.10.008

[45] Lauerman LH. Mycoplasma PCR Assays. In: Lauerman, L.H. (Ed.), Nucleic Acid Amplification Assays for Diagnosis of Animal Diseases. American Association of Veterinary Laboratory Diagnosticians, Auburn, Alabama, pp. 41–42.

[46] Marois C, DufourGesbert F, Kempf I. Detection of *Mycoplasma synoviae* in poultry environment samples by culture and polymerase chain reaction. Vet Microbiol. 2000;73(4):311–8. 10781729 10.1016/s0378-1135(00)00178-4

[47] Raviv Z, Kleven SH. The development of diagnostic real-time TaqMan PCRs for the four pathogenic avian Mycoplasmas. Avian Dis. 2009;53(1):103–7. 19432011 10.1637/8469-091508-Reg.1

[48] Sprygin AV, Andreychuk DB, Kolotilov AN, Volkov MS, Runina IA, Mudrak NS, et al. Development of a duplex real-time TaqMan PCR assay with an internal control for the detection of Mycoplasma gallisepticum and Mycoplasma synoviae in clinical samples from commercial and backyard poultry. Avian Pathol. 2010;39(2):99–109. 20390544 10.1080/03079451003604621

[49] Wang H, Fadl AA, Khan MI. Multiplex PCR for avian pathogenic Mycoplasmas. Mol Cell Probes. 1997;11(3):211–6. 9232620 10.1006/mcpr.1997.0108

[50] Nascimento ER, Yamamoto R, Herrick KR, Tait RC. Polymerase chain reaction for detection of *Mycoplasma gallisepticum*. Avian Dis. 1991;35(1):62–9. 2029263

[51] Verdin E, Saillard C, Labbe A, Bove JM, Kobisch M. A nested PCR assay for the detection of *Mycoplasma hyopneumoniae* in tracheobronchiolar washings from pigs. Vet Microbiol. 2000;76(1):31–40. 10925039 10.1016/s0378-1135(00)00228-5

[52] Marois C, Dory D, Fablet C, Madec F, Kobisch M. Development of a quantitative Real-Time TaqMan PCR assay for determination of the minimal dose of *Mycoplasma hyopneumoniae* strain 116 required to induce pneumonia in SPF pigs. J Appl Microbiol. 2010;108(5):1523–33. 19811567 10.1111/j.1365-2672.2009.04556.x

[53] Fourour S, Fablet C, Tocqueville V, Dorenlor V, Eono F, Eveno E, et al. A new multiplex real-time TaqMan((R)) PCR for quantification of Mycoplasma hyopneumoniae, M. hyorhinis and M. flocculare: exploratory epidemiological investigations to research mycoplasmal association in enzootic pneumonia-like lesions in slaughtered pigs. J Appl Microbiol. 2018;125(2):345–55. 29603531 10.1111/jam.13770

[54] Mattsson JG, Bergstrom K, Wallgren P, Johansson KE. Detection of *Mycoplasma hyopneumoniae* in nose swabs from pigs by in vitro amplification of the 16S rRNA gene. J Clin Microbiol. 1995;33(4):893–7. 7540629 10.1128/jcm.33.4.893-897.1995 PMC228062

[55] Wu Y, Ishag H, Hua L, Zhang L, Liu B, Zhang Z, et al. Establishment and application of a real-time, duplex PCR method for simultaneous detection of *Mycoplasma hyopneumoniae* and *Mycoplasma hyorhinis*. Kafkas Univ Vet Fak Derg. 2019;25(3):405–14.

[56] Foldi D, Nagy ZE, Belecz N, Szeredi L, Foldi J, Kollar A, et al. Establishment of a *Mycoplasma hyorhinis* challenge model in 5-week-old piglets. Front Microbiol. 2023;14:1209119. 37601388 10.3389/fmicb.2023.1209119 PMC10436309

[57] Jeffery N, Gasser RB, Steer PA, Noormohammadi AH. Classification of *Mycoplasma synoviae* strains using single-strand conformation polymorphism and high-resolution melting-curve analysis of the VlhA gene single-copy region. Microbiol (Reading). 2007;153(Pt 8):2679–88. 10.1099/mic.0.2006/005140-0 17660432

[58] McAuliffe L, Ellis RJ, Ayling RD, Nicholas RA. Differentiation of Mycoplasma species by 16S ribosomal DNA PCR and denaturing gradient gel electrophoresis fingerprinting. J Clin Microbiol. 2003;41(10):4844–7. 14532239 10.1128/JCM.41.10.4844-4847.2003 PMC254308

[59] McAuliffe L, Ellis RJ, Lawes JR, Ayling RD, Nicholas RA. 16S rDNA PCR and denaturing gradient gel electrophoresis; a single generic test for detecting and differentiating *Mycoplasma species*. J Med Microbiol. 2005;54(Pt 8):731–9. 16014426 10.1099/jmm.0.46058-0

[60] Deeney AS, Collins R, Ridley AM. Identification of *Mycoplasma* species and related organisms from ruminants in England and Wales during 2005–2019. BMC Vet Res. 2021;17(1):325. 34641885 10.1186/s12917-021-03037-y PMC8513359

[61] Pereyre S, Tardy F, Renaudin H, Cauvin E, Del Pra Netto Machado L, Tricot A, et al. Identification and subtyping of clinically relevant human and ruminant Mycoplasmas by use of matrix-assisted laser desorption ionization-time of flight mass spectrometry. J Clin Microbiol. 2013;51(10):3314–23. 23903545 10.1128/JCM.01573-13 PMC3811644

[62] Spergser J, Hess C, Loncaric I, Ramirez AS. Matrix-Assisted laser desorption Ionization-Time of flight mass spectrometry is a superior diagnostic tool for the identification and differentiation of Mycoplasmas isolated from animals. J Clin Microbiol. 2019;57(9):e00316–19. 10.1128/JCM.00316-19 PMC6711924 31217275

[63] Bokma J, Pardon B, Van Driessche L, Gille L, Deprez P, Haesebrouck F, et al. Optimizing identification of *Mycoplasma Bovis* by MALDI-TOF MS. Res Vet Sci. 2019;125:185–8. 31252368 10.1016/j.rvsc.2019.06.010

[64] Baudler L, Scheufen S, Ziegler L, Moller Palau-Ribes F, Ewers C, Lierz M. Identification and differentiation of avian *Mycoplasma* species using MALDI-TOF MS. J Vet Diagn Invest. 2019;31(4):620–4. 31184287 10.1177/1040638719856932 PMC6857029

[65] Poumarat F, Perrin B, Longchambon D. Identification of ruminant Mycoplasma by dot-immunobinding on membrane filtration (MF dot). Vet Microbiol. 1991;29:329–38. 1771755 10.1016/0378-1135(91)90140-b

[66] Rosendal S, Black FT. Direct and indirect Immunofluorescence of unfixed and fixed Mycoplasma colonies. Acta Pathol Microbiol Scand B Microbiol Immunol. 1972;80(4):615–22. 4566183 10.1111/j.1699-0463.1972.tb00186.x

[67] WOAH. Avian mycoplasmosis (*Mycoplasma gallisepticum*, *M. synoviae*) 2021. In: WOAH Terrestrial Manual [Internet].

