# Supplementary material for: Advancing standardization of diagnostics and antimicrobial susceptibility testing for pathogenic mycoplasmas of livestock origin: insights from the MyMIC network
Source: BMC Vet Res. 2025 Dec 29;21:712. doi: 10.1186/s12917-025-05154-4 (PMC12751987; doi:10.1186/s12917-025-05154-4)
Supplement: Supplementary file 1 — Additional file 1.pdf: Text of the survey on field practices regarding diagnosis and antimicrobial use for controlling mycoplasmoses as sent out to veterinarians [file 12917_2025_5154_MOESM1_ESM.pdf]

Mycoplasma questionnaire

You will find on this page the questionnaire for you to answer on **antimicrobials that are used for treatment of Mycoplasma infections in different livestocks sectors.** We thank you for the interest you put into our work and your help as respondents.

This questionnaire is no longer available. Thank you for your time and responses.

Please select the language you feel the most comfortable with to answer the questionnaire about your practices :

- English
- French
- Dutch
- Hungarian
- Finnish
- Italian
- German
- Swedish
- Spanish
- Portuguese

NB : If your language is not available, please contact us (see below).

Following are the institutions taking part in the project :

Contact : Claire Becker (claire.becker@vetagro-sup.fr) and Jeanine Wiegel (j.wiegel@gdanimalhealth.com)

Antimicrobial use on mycoplasmoses in livestock

Target respondents : **Veterinary practitioners in livestock and poultry**.
This questionnaire is part of a work from a network funded by **JPI AMR** on veterinary Mycoplasma's (MyMIC network: https://www.jpiamr.eu/projects/mymic/).
The name of this network is "**Standardization of diagnostics and antimicrobial susceptibility testing and clinical interpretation in animal mycoplasmas**". The objective of this project is to set up a network of laboratories working on mycoplasma diagnostics and their susceptibility to antibiotics to compare the different methods used and the results of minimum inhibitory concentrations (MIC).Within one of the work packages we would like to collect data on **antimicrobials that are used for treatment of Mycoplasma infections** in different livestock sectors. Data on antimicrobial use is often only available on a national level, without information on the aetiology. Therefore, we would like to ask practitioners about the antibiotic use related to Mycoplasma infections in livestock through this questionnaire.

We kindly ask you, as a vet practitioner, to answer to the following questions, regarding your **real practices in the field**. The analysis of data will be **anonymous** and will be **published in scientific international journals**. If you have any concern about this questionnaire, do not hesitate to contact us at claire.becker@vetagro-sup.fr or j.wiegel@gdanimalhealth.com.

Please answer this questionnaire until the end once you started it.
Duration time : around 15 minutes.

In which country do you work as a veterinary practitioner? *

- Afghanistan

How many years of experience do you have as a veterinary practitioner? *

- (•) <5 years
- ( ) 6-15 years
- ( ) 16-25 years
- ( ) >25 years

In which type of veterinary practice do you work? *

- [x] Mixed practice
- [x] Specialised in pigs
- [x] Specialised in poultry
- [x] Specialised in cattle
- [ ] Other :

How many livestock veterinarians work in your practice? *

- (•) 1 to 3
- ( ) 4 to 6
- ( ) 7 to 9
- ( ) over 10

What are the main livestock species you work with?
Follow up questions will appear related to the species that are selected.
Multiple species are possible. *

- [x] dairy cattle
- [x] cattle rearing
- [x] beef cattle
- [x] pig nursery
- [x] weaning piglets
- [x] fattening pigs
- [x] breeding sows
- [x] chickens - layers
- [x] chickens - breeders
- [x] chickens - broilers
- [ ] meat turkeys
- [ ] other :

Do you encounter Mycoplasma infections in the animals you see? *

- (•) Yes
- ( ) No

If yes, select the animal species. *

- [x] dairy cattle
- [x] cattle rearing
- [x] beef cattle
- [x] pig nursery
- [x] weaning piglets
- [x] fattening pigs
- [x] breeding sows
- [x] chickens - layers
- [x] chickens - breeders
- [x] chickens - broilers
- [ ] meat turkeys
- [ ] other :

[BOVINE]

[Dairy cattle] What Mycoplasma species do you encounter related to clinical disease? *

- [x] M. bovis
- [ ] Don't know
- [ ] Other :

[Cattle rearing] What Mycoplasma species do you encounter related to clinical

- [x] M. bovis

disease? *

- [ ] Don't know
- [ ] Other :

[Beef cattle] What Mycoplasma species do you encounter related to clinical disease? *

- [x] M. bovis
- [ ] Don't know
- [ ] Other :

[Bovine] What is the proportion of animals affected when you encounter a clinical infection with Mycoplasma?

- ( ) Less than 10%
- (•) 10 to 25%
- ( ) 25 to 50%
- ( ) 50 to 75%
- ( ) 75 to 100%

[Bovine] Are additional diagnostics applied when a Mycoplasma infection is suspected (on the differential diagnosis)?

- ( ) never (=0)
- ( ) rarely (<1/10)
- (•) occasionally (1/10 to 1/2)
- ( ) often (> 1/2)
- ( ) always (>4/5)

[Bovine] If yes, what additional diagnostics? *

- [ ] Serology
- [ ] PCR
- [x] Culture
- [ ] Post mortem (necropsy)
- [ ] Histology
- [ ] Other :

[Bovine] Do you perform (or let a third party perform) antimicrobial susceptibility tests? *

- ( ) never (=0)
- ( ) rarely (<1/10)
- (•) occasionally (1/10 to 1/2)
- ( ) often (> 1/2)
- ( ) always (>4/5)

[Bovine] Is treatment required for Mycoplasma infections? *

- ( ) never (=0)
- ( ) rarely (<1/10)
- (•) occasionally (1/10 to 1/2)
- ( ) often (> 1/2)
- ( ) always (>4/5)

[Bovine] When do you decide to apply treatment for Mycoplasma on diseased animals ? *

- [ ] based on the severity of signs and the most likely diagnosis
- [ ] results of further laboratory testing
- [x] known disease which spreads quickly
- [ ] percentage of animals diseased
- [ ] request from farmer/industry
- [ ] other:

[Bovine] Do you perform metaphylactic treatment for Mycoplasma infections? *

- ( ) never (=0)
- ( ) rarely (<1/10)
- (•) occasionally (1/10 to 1/2)
- ( ) often (> 1/2)
- ( ) always (>4/5)

[Bovine] What is the reason that makes you decide to apply metaphylactic treatment on a lot ? *

- [ ] severity of signs
- [ ] results of laboratory tests
- [x] known disease which spreads quickly
- [ ] percentage of animals diseased
- [ ] request from farmer/industry
- [ ] other:

[Bovine] If you decide not to treat for Mycoplasma infections, why is that? *

- [ ] Expected lack of efficacy
- [ ] Impact of treatment on trade / Due to industry agreements
- [x] Legal boundaries
- [ ] Logistic problems due to withdrawal times
- [ ] Cost
- [ ] Other :

[Bovine] What is the preferred option of treatment on Mycoplasma infections? (only answer for the species that you treat)

	fluoroquinolones	macrolides	pleuromutilins	lincosamides	cephalosporins	aminoglycosides	florfenicol	penicillins without betalactam inhibitors	penicillins with betalactam inhibitors	tetracyclines	trimethoprim / sulfonamides	combinations	long-acting forms	no treatment
dairy cattle	( )	( )	( )	( )	( )	( )	( )	( )	( )	( )	( )	(•)	( )	( )

cattle rearing	○	○	○	○	○	○	○	○	○	○	○	○	◉	○
beef cattle	○	○	○	○	○	○	○	○	○	○	○	○	○	◉

[Bovine] If you ticked combinations, please precise the molecules :

,

[Bovine] If you ticked long-acting forms, please precise the molecules :

,

[Bovine] If you ticked no treatment, please precise why :

,

[Bovine] Concerning the preferred treatment option selected, what dose and duration do you generally apply ? If you selected multiple options, please precise the molecules. *

,

[Bovine] What is your preferred treatment option based on? *

- [ ] National treatment guidelines
- [x] Farm History
- [ ] Antimicrobial Susceptibility Testing results
- [ ] Overviews of Antimicrobial Susceptibility of the agent
- [ ] Economic feasability of treatment
- [ ] Other :

[Bovine] How often do you use the following antimicrobial classes for treatment of Mycoplasma infections? (only answer for the species that you treat)	never (=0)	rarely (<1/10)	occasionally (1/10 to 1/2)	often (> 1/2)	always (>4/5)
fluoroquinolones	○	◉	○	○	○

macrolides	○	○	●	○	○
pleuromutilins	○	○	○	●	○
lincosamides	○	○	●	○	○
cephalosporins°	○	●	○	○	○
aminoglycosides	○	●	○	○	○
florfenicol	○	●	○	○	○
penicillins without betalactam inhibitors°	○	●	○	○	○
penicillins with betalactam inhibitors°	○	●	○	○	○
tetracyclines	○	●	○	○	○
trimethoprim/sulfonamides	○	●	○	○	○
long-acting forms	○	●	○	○	○
combinations	○	●	○	○	○

°NOTE: cephalosporins and penicillins are not effective in treating Mycoplasma. Please explain in the textbox why you have selected this option :

;

[Bovine] If you ticked combinations, please precise the molecules :

;

[Bovine] If you ticked long-acting forms, please precise the molecules :

;

[Bovine] What is the most frequent pattern for metaphylactic treatment that you apply in your practice ? Tick the corresponding boxes - antibiotic and administration route	per os / premixed feed	per os / feed top dressing	per os / drinking water	parenteral / subcutaneous	parenteral / intramuscular	parenteral / intravenous	other
fluoroquinolones	◉	○	○	○	○	○	○
macrolides	◉	○	○	○	○	○	○
pleuromutilins	◉	○	○	○	○	○	○
lincosamides	◉	○	○	○	○	○	○
cephalosporins°	◉	○	○	○	○	○	○
aminoglycosides	◉	○	○	○	○	○	○
florfenicol	◉	○	○	○	○	○	○
penicillins without betalactam inhibitors°	◉	○	○	○	○	○	○
penicillins with betalactam inhibitors°	◉	○	○	○	○	○	○

tetracyclines	(•)	( )	( )	( )	( )	( )	( )
trimethoprim/sulfonamides	(•)	( )	( )	( )	( )	( )	( )
long-acting forms	(•)	( )	( )	( )	( )	( )	( )
combinations	(•)	( )	( )	( )	( )	( )	( )

°NOTE: cephalosporins and penicillins are not effective in treating Mycoplasma. Please explain in the textbox why you have selected this option :

,

[Bovine] If you ticked combinations, please precise the molecules :

,

[Bovine] If you ticked long-acting forms, please precise the molecules :

,

[Bovine] In case of failure of treatment, what is the second choice of treatment available?

- ( ) fluoroquinolones
- ( ) macrolides
- ( ) pleuromutilins
- ( ) lincosamides
- ( ) cephalosporins
- ( ) aminoglycosides

- ( ) florfenicol
- ( ) penicillins without betalactam inhibitors
- ( ) penicillins with betalactam inhibitors
- ( ) tetracyclines
- ( ) trimethoprim/sulfonamides
- (•) combinations (write the molecules below)
- ( ) long-acting forms (precise below)
- ( ) no treatment, because:

[Bovine] If you ticked combinations, please precise the molecules :

;

[PORCINE]

[Pig nursery] What Mycoplasma species do you encounter related to clinical disease? *

- [x] M. hyopneumoniae
- [ ] M. hyorhinis
- [ ] Don't know
- [ ] Other :

[Weaning piglets] What Mycoplasma species do you encounter related to clinical disease? *

- [ ] M. hyopneumoniae
- [x] M. hyorhinis
- [ ] Don't know
- [ ] Other :

[Fattening pigs] What Mycoplasma species do you encounter related to clinical disease? *

- [ ] M. hyopneumoniae
- [x] M. hyorhinis
- [ ] Don't know
- [ ] Other :

[Breeding sows] What Mycoplasma species do you encounter related to clinical disease? *

- [ ] M. hyopneumoniae
- [x] M. hyorhinis
- [ ] Don't know

- [ ] Other :

[Porcine] What is the proportion of animals affected when you encounter a clinical infection with Mycoplasma?

- ( ) Less than 10%
- ( ) 10 to 25%
- (•) 25 to 50%
- ( ) 50 to 75%
- ( ) 75 to 100%

[Porcine] Are additional diagnostics applied when a Mycoplasma infection is suspected (on the differential diagnosis)?

- ( ) never (=0)
- ( ) rarely (<1/10)
- (•) occasionally (1/10 to 1/2)
- ( ) often (> 1/2)
- ( ) always (>4/5)

[Porcine] If yes, what additional diagnostics? *

- [ ] Serology
- [ ] PCR
- [x] Culture
- [ ] Post mortem (necropsy)
- [ ] Histology
- [ ] Other :

[Porcine] Do you perform (or let a third party perform) antimicrobial susceptibility tests? *

- ( ) never (=0)
- ( ) rarely (<1/10)
- (•) occasionally (1/10 to 1/2)
- ( ) often (> 1/2)
- ( ) always (>4/5)

[Porcine] Is treatment required for Mycoplasma infections? *

- ( ) never (=0)
- ( ) rarely (<1/10)
- (•) occasionally (1/10 to 1/2)
- ( ) often (> 1/2)
- ( ) always (>4/5)

[Porcine] When do you decide to apply treatment for Mycoplasma on diseased animals ? *

- [ ] based on the severity of signs and the most likely diagnosis
- [ ] results of further laboratory testing
- [x] known disease which spreads quickly
- [ ] percentage of animals diseased

- [ ] request from farmer/industry
- [ ] other:

[Porcine] Do you perform metaphylactic treatment for Mycoplasma infections? *

- ( ) never (=0)
- ( ) rarely (<1/10)
- (•) occasionally (1/10 to 1/2)
- ( ) often (> 1/2)
- ( ) always (>4/5)

[Porcine] What is the reason that makes you decide to apply metaphylactic treatment on a lot ? *

- [ ] severity of signs
- [ ] results of laboratory tests
- [x] known disease which spreads quickly
- [ ] percentage of animals diseased
- [ ] request from farmer/industry
- [ ] other:

[Porcine] If you decide not to treat for Mycoplasma infections, why is that? *

- [ ] Expected lack of efficacy
- [ ] Impact of treatment on trade / Due to industry agreements
- [x] Legal boundaries
- [ ] Logistic problems due to withdrawal times
- [ ] Cost
- [ ] Other :

[Porcine] What is the preferred option of treatment on Mycoplasma infections? (only answer for the species that you treat)

	fluoroquinolones	macrolides	pleuromutilins	lincosamides	cephalosporins	aminoglycosides	florfenicol	penicillins without betalactam inhibitors	penicillins with betalactam inhibitors	tetracyclines	trimethoprim / sulfonamides	combinations	long-acting forms	no treatment
nursery pigs	○	○	○	○	○	○	○	○	○	○	○	●	○	○
weaning piglets	○	○	○	○	○	○	○	○	○	○	○	○	●	○
fattening pigs	○	○	○	○	○	○	○	○	○	○	○	○	○	●
breeding sows	○	○	○	○	○	○	○	○	○	○	○	●	○	○

[Porcine] If you ticked combinations, please precise the molecules :

;

[Porcine] If you ticked long-acting forms, please precise the molecules :

;

[Porcine] If you ticked no treatment, please precise why :

;;

[Porcine] Concerning the preferred treatment option selected, what dose and duration do you generally apply ? If you selected multiple options, please precise the molecules. *

;

[Porcine] What is your preferred treatment option based on? *

- [ ] National treatment guidelines
- [ ] Farm History
- [x] Antimicrobial Susceptibility Testing results
- [ ] Overviews of Antimicrobial Susceptibility of the agent
- [ ] Economic feasability of treatment
- [ ] Other :

[Porcine] How often do you use the following antimicrobial classes for treatment of Mycoplasma infections? (only answer for the species that you treat)

	never (=0)	rarely (<1/10)	occasionally (1/10 to 1/2)	often (> 1/2)	always (>4/5)
fluoroquinolones	○	●	○	○	○
macrolides	○	●	○	○	○
pleuromutilins	○	●	○	○	○
lincosamides	○	●	○	○	○

cephalosporins°	○	◉	○	○	○
aminoglycosides	○	◉	○	○	○
florfenicol	○	◉	○	○	○
penicillins without betalactam inhibitors°	○	◉	○	○	○
penicillins with betalactam inhibitors°	○	◉	○	○	○
tetracyclines	○	◉	○	○	○
trimethoprim/sulfonamides	○	◉	○	○	○
long-acting forms	○	◉	○	○	○
combinations	○	◉	○	○	○

°NOTE: cephalosporins and penicillins are not effective in treating Mycoplasma. Please explain in the textbox why you have selected this option :

;

[Porcine] If you ticked combinations, please precise the molecules :

;

[Porcine] If you ticked long-acting forms, please precise the molecules :

;

[Porcine] What is the most frequent pattern for metaphylactic treatment that you apply in your practice ? Tick the corresponding boxes - antibiotic and administration route	per os / premixed feed	per os / feed top dressing	per os / drinking water	parenteral / subcutaneous	parenteral / intramuscular	parenteral / intravenous	other
fluoroquinolones	X						
macrolides	X						
pleuromutilins	X						
lincosamides	X						
cephalosporins°	X						
aminoglycosides	X						
florfenicol	X						
penicillins without betalactam inhibitors°	X						
penicillins with betalactam inhibitors°	X						
tetracyclines	X						

trimethoprim/sulfonamides	(•)	( )	( )	( )	( )	( )	
long-acting forms	(•)	( )	( )	( )	( )	( )	( )
combinations	(•)	( )	( )	( )	( )	( )	( )

°NOTE: cephalosporins and penicillins are not effective in treating Mycoplasma. Please explain in the textbox why you have selected this option :

;

[Porcine] If you ticked combinations, please precise the molecules :

;

[Porcine] If you ticked long-acting forms, please precise the molecules :

:

[Porcine] In case of failure of treatment, what is the second choice of treatment available?

- ( ) fluoroquinolones
- ( ) macrolides
- ( ) pleuromutilins
- ( ) lincosamides
- ( ) cephalosporins
- ( ) aminoglycosides
- ( ) florfenicol
- ( ) penicillins without betalactam inhibitors
- ( ) penicillins with betalactam inhibitors

- ( ) tetracyclines
- ( ) trimethoprim/sulfonamides
- ( ) combinations (write the molecules below)
- (•) long-acting forms (precise below)
- ( ) no treatment, because:

[Porcine] If you ticked long-acting forms, please precise the molecules :

;

[POULTRY]

[Chickens - layers] What Mycoplasma species do you encounter related to clinical disease? *

- [ ] M. gallisepticum
- [x] M. synoviae
- [ ] Don't know
- [ ] Other :

[Chickens - breeders] What Mycoplasma species do you encounter related to clinical disease? *

- [ ] M. gallisepticum
- [x] M. synoviae
- [ ] Don't know
- [ ] Other :

[Chickens - broilers] What Mycoplasma species do you encounter related to clinical disease? *

- [ ] M. gallisepticum
- [x] M. synoviae
- [ ] Don't know
- [ ] Other :

[Poultry] What is the proportion of animals affected when you encounter a clinical infection with Mycoplasma?

- ( ) Less than 10%
- ( ) 10 to 25%
- (•) 25 to 50%
- ( ) 50 to 75%
- ( ) 75 to 100%

[Poultry] Are additional diagnostics applied when a Mycoplasma infection is

- ( ) never (=0)

suspected (on the differential diagnosis)?

- ○ rarely (<1/10)
- ● occasionally (1/10 to 1/2)
- ○ often (> 1/2)
- ○ always (>4/5)

[Poultry] If yes, what additional diagnostics? *

- [ ] Serology
- [ ] PCR
- [x] Culture
- [ ] Post mortem (necropsy)
- [ ] Histology
- [ ] Other :

[Poultry] Do you perform (or let a third party perform) antimicrobial susceptibility tests? *

- ○ never (=0)
- ○ rarely (<1/10)
- ● occasionally (1/10 to 1/2)
- ○ often (> 1/2)
- ○ always (>4/5)

[Poultry] Is treatment required for Mycoplasma infections? *

- ○ never (=0)
- ○ rarely (<1/10)
- ● occasionally (1/10 to 1/2)
- ○ often (> 1/2)
- ○ always (>4/5)

[Poultry] When do you decide to apply treatment for Mycoplasma on diseased animals ? *

- [ ] based on the severity of signs and the most likely diagnosis
- [ ] results of further laboratory testing
- [x] known disease which spreads quickly
- [ ] percentage of animals diseased
- [ ] request from farmer/industry
- [ ] other:

[Poultry] Do you perform metaphylactic treatment for Mycoplasma infections? *

- ○ never (=0)
- ○ rarely (<1/10)
- ● occasionally (1/10 to 1/2)
- ○ often (> 1/2)
- ○ always (>4/5)

[Poultry] What is the reason that makes you decide to apply metaphylactic treatment on a lot ? *

- [ ] severity of signs
- [ ] results of laboratory tests
- [x] known disease which spreads quickly
- [ ] percentage of animals diseased
- [ ] request from farmer/industry
- [ ] other:

[Poultry] If you decide not to treat for Mycoplasma infections, why is that? *

- [ ] Expected lack of efficacy
- [ ] Impact of treatment on trade / Due to industry agreements
- [x] Legal boundaries
- [ ] Logistic problems due to withdrawal times
- [ ] Cost
- [ ] Other :

[Poultry] What is the preferred option of treatment on Mycoplasma infections? (only answer for the species that you treat)

	fluoroquinolones	macrolides	pleuromutilins	lincosamides	cephalosporins	aminoglycosides	florfenicol	penicillins without betalactam inhibitors	penicillins with betalactam inhibitors	tetracyclines	trimethoprim / sulfonamides	combinations	long-acting forms	no treatment
chickens - layers	( )	( )	( )	( )	( )	( )	( )	( )	( )	( )	( )	(•)	( )	( )
chickens - breeders	( )	( )	( )	( )	( )	( )	( )	( )	( )	( )	( )	( )	(•)	( )
chickens - broilers	( )	( )	( )	( )	( )	( )	( )	( )	( )	( )	( )	( )	( )	(•)
meat turkeys	( )	( )	( )	( )	( )	( )	( )	( )	( )	( )	( )	(•)	( )	( )

[Poultry] If you ticked combinations, please precise the molecules :

;

[Poultry] If you ticked long-acting forms, please precise the

molecules :

;

[Poultry] If you ticked no treatment, please precise why :

;

[Poultry] Concerning the preferred treatment option selected, what dose and duration do you generally apply ? If you selected multiple options, please precise the molecules. *

;

[Poultry] What is your preferred treatment option based on? *

- [ ] National treatment guidelines
- [ ] Farm History
- [ ] Antimicrobial Susceptibility Testing results
- [x] Overviews of Antimicrobial Susceptibility of the agent
- [ ] Economic feasability of treatment
- [ ] Other :

[Poultry] How often do you use the following antimicrobial classes for treatment of Mycoplasma infections? (only answer for the species that you treat)

	never (=0)	rarely (<1/10)	occasionally (1/10 to 1/2)	often (> 1/2)	always (>4/5)
fluoroquinolones	○	●	○	○	○
macrolides	○	●	○	○	○
pleuromutilins	○	●	○	○	○
lincosamides	○	●	○	○	○
cephalosporins°	○	●	○	○	○
aminoglycosides	○	●	○	○	○
florfenicol	○	●	○	○	○

penicillins without betalactam inhibitors°	○	◉	○	○	○
penicillins with betalactam inhibitors°	○	◉	○	○	○
tetracyclines	○	◉	○	○	○
trimethoprim/sulfonamides	○	◉	○	○	○
long-acting forms	○	◉	○	○	○
combinations	○	◉	○	○	○

°NOTE: cephalosporins and penicillins are not effective in treating Mycoplasma. Please explain in the textbox why you have selected this option :

,

[Poultry] If you ticked combinations, please precise the molecules :

,

[Poultry] If you ticked long-acting forms, please precise the molecules :

,

[Poultry] What is the most frequent pattern for metaphylactic treatment that you apply in your practice ? Tick the corresponding boxes - antibiotic and administration route	per os / premixed feed	per os / feed top dressing	per os / drinking water	parenteral / subcutaneous	parenteral / intramuscular	parenteral / intravenous	other
fluoroquinolones	◉	○	○	○	○	○	○
macrolides							○

pleuromutilins

lincosamides

cephalosporins°

aminoglycosides

florfenicol

penicillins without betalactam inhibitors°

penicillins with betalactam inhibitors°

tetracyclines

trimethoprim/sulfonamides

long-acting forms

combinations

°NOTE: cephalosporins and penicillins are not effective in treating Mycoplasma. Please explain in the textbox why you have selected this option :

n

[Poultry] If you ticked combinations, please precise the molecules :

n

[Poultry] If you ticked long-acting forms, please precise the molecules :

n

[Poultry] In case of failure of treatment, what is the second choice of treatment available?

- ( ) fluoroquinolones
- ( ) macrolides
- ( ) pleuromutilins
- ( ) lincosamides
- ( ) cephalosporins
- ( ) aminoglycosides
- ( ) florfenicol
- ( ) penicillins without betalactam inhibitors
- ( ) penicillins with betalactam inhibitors
- ( ) tetracyclines
- ( ) trimethoprim/sulfonamides
- ( ) combinations (write the molecules below)
- (•) long-acting forms (precise below)
- ( ) no treatment, because:

[Poultry] If you ticked long-acting forms, please precise the molecules :

,

[OTHER]

If you ticked other, please precise the animal species and the Mycoplasma species : *

If you use treatment for these species, please precise the molecules and the administration route : *

If you have additional information on treatments of Mycoplasma infections, please share it here

;

If you would like to receive a summary of the survey outcome (during the second part of 2024), please fill in your email address here. We will contact you as soon as the report is ready.

;

* = Input is required
