# Supplementary material for: Advancing standardization of diagnostics and antimicrobial susceptibility testing for pathogenic mycoplasmas of livestock origin: insights from the MyMIC network
Source: BMC Vet Res. 2025 Dec 29;21:712. doi: 10.1186/s12917-025-05154-4 (PMC12751987; doi:10.1186/s12917-025-05154-4)
Supplement: Supplementary file 2 — Additional file 2.pdf: Text of the survey of the current practices in terms of culture, identification and AST as filled in by partners’ laboratories [file 12917_2025_5154_MOESM2_ESM.pdf]

Questionnaire MyMIC 2023

Dear colleagues,

Thank you for participating in the MyMIC Working Package 1 (WP1) questionnaire aiming at:

- Collecting information on methodologies used in partners' laboratories for culture, identification, and antimicrobial susceptibility testing (AST) of animal mycoplasmas
- Evaluating availability of AST data among the partners obtained using harmonized methods
- Evaluating availability of “reference” strains in partners' archive

This questionnaire will only be shared among MyMIC participants. Please note that the data from this questionnaire will be processed by the WP1 of the MyMIC network.

The questionnaire is designed to record information:

- **per institute**
- regarding **only one mycoplasma species at a time**.

Please **make sure all the mycoplasma species being investigated at your institute have been covered by filling one questionnaire per species**.

Please feel free to distribute the questionnaire among your team and nominate one person for one (or more) mycoplasma species.

We estimate that filling the questionnaire for each mycoplasma species will take 15 minutes maximum.

Thanks in advance for your participation before **DATE**

MyMIC WP1 team

Questionnaire MyMIC 2023

Preliminary questions

Your institute

Country :

Enter your text here

Institution name :

Enter your text here

Your details

Family name :

Enter your text here

First name :

Enter your text here

E-mail :

Previous | Next

Preliminary questions

Which mycoplasma species will be covered in this questionnaire ?

Please fill one questionnaire per institution and per mycoplasma species.

- ◯ M. hyopneumoniae
- ◯ M. hyorhinis
- ◯ M. synoviae
- ◯ M. gallisepticum
- ◯ M. bovis
- ◯ None of the previous listed species (i.e. "additionnal species")

Preliminary questions

Please specify on which of the following "additionnal species" your institution is working :

- ◯ M. hyosynoviae
- ◯ M. mycoides subsp. mycoides
- ◯ M. dispar
- ◯ M. capricolum subsp. capripneumoniae
- ◯ M. agalactiae
- ◯ M. mycoides subsp. capri
- ◯ M. mycoides subsp. capricolum
- ◯ M. ovipneumoniae
- ◯ M. putrefaciens
- ◯ Other

please specify

Thank you for your time, the WP5 team dedicated to the "additional mycoplasma species" will be in touch for follow up questions.

Previous Next

Culture

Please answer for one medium at a time.

Name of culture medium used :

Enter your text here

Form of medium used

- ◯ Agar
- ◯ Broth
- ◯ Both agar and broth

Preparation of the medium :

- ☐ commercial
- ☐ in-house

Supplier of the medium :

Enter your text here

Incubation temperature :

Enter your text here

Incubation atmosphere :

Enter your text here

Detection of growth in broth :

- ☐ turbidity (naked eye)
- ☐ pH indicator
- ☐ Other

Please specify

Previous Next

Culture

Is another medium used for this species in your institution?

- ◯ Yes
- ◯ No

Culture

Please answer for one medium at a time.

Name of culture medium used :

Enter your text here

Form of medium used

- ◯ Agar
- ◯ Broth
- ◯ Both agar and broth

Preparation of the medium :

- ☐ commercial
- ☐ in-house

Supplier of the medium :

Enter your text here

Incubation temperature :

Enter your text here

Incubation atmosphere :

Enter your text here

Detection of growth in broth :

- ☐ turbidity (naked eye)
- ☐ pH indicator
- ☐ Other

Please specify

Culture

Is another medium used for this species in your institution?

- ◯ Yes
- ◯ No

Previous Next

Culture

Please answer for one medium at a time.

Name of culture medium used :

Enter your text here

Form of medium used

- ◯ Agar
- ◯ Broth
- ◯ Both agar and broth

Preparation of the medium :

- ☐ commercial
- ☐ in-house

Supplier of the medium :

Enter your text here

Incubation temperature :

Enter your text here

Incubation atmosphere :

Enter your text here

Detection of growth in broth :

- ☐ turbidity (naked eye)
- ☐ pH indicator
- ☐ Other

Please specify

Previous Next

Culture

Quantification of mycoplasma load :

- [ ] Colony count in agar (CFU)
- [ ] Color Changing Units (CCU) in broth
- [ ] Other

Please specify

Further comments regarding quantification methods :

Enter your text here

Previous Next

Culture

Further comments regarding culture :

Enter your text here

Previous Next

Identification of isolates

To what level do you identify the isolates ?

- [ ] species or subspecies
- [ ] strain (e.g. vaccine strain, wildtype,...)
- [ ] subtypes (molecular pattern)

Please select the identification method(s) used for strains enlisted in AST :

Please select mainly used methods. Several options can be selected.

- [ ] conventional PCR
- [ ] real-time PCR
- [ ] DGGE PCR
- [ ] immunobased method (e.g. immunofluorescence, growth inhibition, dot-immunobinding,...)
- [ ] whole genome sequencing
- [ ] Other

Please specify any missing method(s) __________

Previous | Next

Identification of isolates - conventional PCR

Please provide a short description of the conventional PCR(s) used or a reference article :

Enter your text here

Please specify preliminary steps of the conventional PCR used :

e.g. culture, cloning, DNA extraction, boiling, …

Enter your text here

Please provide details on the control(s) used for this method :

Enter your text here

Do you differentiate between vaccine and clinical strains ?

◯ Yes

◯ No

Please provide a short description of the method used for differenciation of vaccine strain et specify wether it is part of the same identification method or based on complementary test

Enter your text here

Identification of isolates - conventional PCR

Do you identify mixed population of different species ?

◯ Yes

◯ No

Please provide a short description of the method used for differenciation of different species et specify wether it is part of the same identification method or based on complementary test

Enter your text here

Do you identify mixed population of different subtypes ?

◯ Yes

◯ No

Please provide a short description of the method used for differenciation of different subtypes et specify wether it is part of the same identification method or based on complementary test

Enter your text here

Identification of isolates - qPCR

Please provide a short description of the qPCR used or a reference article :

Enter your text here

Please specify preliminary steps of the qPCR used or a reference article :

e.g. culture, cloning, DNA extraction, boiling, …

Enter your text here

Please provide details on the control(s) used for this method :

Enter your text here

Do you differentiate between vaccine and clinical strains ?

- ◯ Yes
- ◯ No

Please provide a short description of the method used for differenciation of vaccine strain et specify wether it is part of the same identification method or based on complementary test

Enter your text here

Identification of isolates - qPCR

Do you identify mixed population of different species ?

◯ Yes

◯ No

Please provide a short description of the method used for differenciation of different species et specify wether it is part of the same identification method or based on complementary test

Enter your text here

Do you identify mixed population of different subtypes ?

◯ Yes

◯ No

Please provide a short description of the method used for differenciation of different subtypes et specify wether it is part of the same identification method or based on complementary test

Enter your text here

Previous Next

Identification of isolates - immunobased method

Please provide a short description of the immunobased method(s) used or a reference article :

Enter your text here

Please specify preliminary steps of the immunobased method(s) used or a reference article :

e.g. cloning, culture of required density

Enter your text here

Please provide details on the control(s) used for this method :

Enter your text here

Do you differentiate between vaccine and clinical strains ?

- ◯ Yes
- ◯ No

Please provide a short description of the method used for differenciation of vaccine strain et specify wether it is part of the same identification method or based on complementary test

Enter your text here

Identification of isolates - immunobased method

Do you identify mixed population of different species ?

◯ Yes

◯ No

Please provide a short description of the method used for differenciation of different species et specify wether it is part of the same identification method or based on complementary test

Enter your text here

Do you identify mixed population of different subtypes ?

◯ Yes

◯ No

Please provide a short description of the method used for differenciation of different subtypes et specify wether it is part of the same identification method or based on complementary test

Enter your text here

Identification of isolates - WGS method

Please provide a short description of the WGS method(s) used or a reference article :

Enter your text here

Please specify preliminary steps of WGS method used or a reference article :

Enter your text here

Please provide details on the control(s) used for this method :

Enter your text here

Do you differentiate between vaccine and clinical strains ?

- ◯ Yes
- ◯ No

Please provide a short description of the method used for differenciation of vaccine strain et specify wether it is part of the same identification method or based on complementary test

Enter your text here

Identification of isolates - WGS method

Do you identify mixed population of different species ?

◯ Yes

◯ No

Please provide a short description of the method used for differenciation of different species et specify wether it is part of the same identification method or based on complementary test

Enter your text here

Do you identify mixed population of different subtypes ?

◯ Yes

◯ No

Please provide a short description of the method used for differenciation of different subtypes et specify wether it is part of the same identification method or based on complementary test

Enter your text here

Identification of isolates - other method

Please provide a short description of other method used or a reference article :

Enter your text here

Please specify preliminary steps of other method used or a reference article :

Enter your text here

Please provide details on the control(s) used for this method :

Enter your text here

Do you differentiate between vaccine and clinical strains ?

- ◯ Yes
- ◯ No

Please provide a short description of the method used for differenciation of vaccine strain et specify wether it is part of the same identification method or based on complementary test

Enter your text here

Previous Next

Identification of isolates - other method

Do you identify mixed population of different species ?

- ◯ Yes
- ◯ No

Please provide a short description of the method used for differenciation of different species et specify wether it is part of the same identification method or based on complementary test

Enter your text here

Do you identify mixed population of different subtypes ?

- ◯ Yes
- ◯ No

Please provide a short description of the method used for differenciation of different subtypes et specify wether it is part of the same identification method or based on complementary test

Enter your text here

Identification of isolates

Further comments regarding identification of isolates :

Enter your text here

Previous Next

Antimicrobial susceptibility testing - phenotypical approaches

Do you perform phenotypical approaches for AST ?

- ◯ Yes
- ◯ No

What method is used in your laboratory ?

- ☐ Agar dilution
- ☐ Broth dilution in tubes
- ☐ Broth dilution in microplates
- ☐ Other

Please specify

Is microplates preparation...

- ☐ Home-made
- ☐ Commercial

Antimicrobial susceptibility testing - agar dilution

What is the medium used for agar dilution method ?

Enter your text here

What is the antimicrobial concentration range used ?

- [ ] Full range
- [ ] truncated range (only several concentrations tested)

Incubation temperature :

Enter your text here

Incubation atmosphere :

Enter your text here

What is the incubation period?

please include start and end of reading if different, expressed in days if possible or indicate the reference used to start/ end the reading e.g. when the growth control shows growth

Enter your text here

Whta is the reading frequency?

Enter your text here

Previous Next

Antimicrobial susceptibility testing - agar dilution

Please indicate your preliminary steps :

How do you prepare the isolates to work with ?

e.g. colony picking, filter cloning…

Enter your text here

How do you preserve the strains for AST?

◯ in broth with glycerol

◯ in broth without glycerol

◯ Other

please specify

Do you calibrate isolates (quantification) before AST assay ?

◯ Yes

◯ No

What is the method used for strain calibration ?

Enter your text here

Antimicrobial susceptibility testing - agar dilution

Assay controls :

What control do you usually include in agar dilution assay ?

- [ ] Positive control : known strain without antimicrobial
- [ ] Positive control : strain with antimicrobial and an expected MIC profile
- [ ] Negative control : sterile media
- [ ] No controls
- [ ] Other

Please specify

Please specify genus/species of the control strains used

Enter your text here

Reading :

What is your reading method ?

- [ ] colony counting using magnifier (manual)
- [ ] automatized colony counting
- [ ] Other

please specify

Previous Next

Antimicrobial susceptibility testing - agar dilution

Validation of the results and interpretation :

How do you validate the results obtained (control results excluded) ?

e.g. colony counts, CCU,...

Enter your text here

What is the number of repetition(s) used to validate a MIC value (replicates in a same run or independent replicates in different run):

Enter your text here

Please indicate the interpretation cut-off used if any :

Enter your text here

Do you have any further comments on agar dilution method ?

Enter your text here

Antimicrobial susceptibility testing - broth dilution in microplates

What is the medium used for agar dilution method ?

Enter your text here

What is the antimicrobial concentration range used ?

- [ ] Full range
- [ ] truncated range (only several concentrations tested)

Incubation temperature :

Enter your text here

Incubation atmosphere :

Enter your text here

What is the incubation period?

please include start and end of reading if different, expressed in days if possible or indicate the reference used to start/ end the reading e.g. when the growth control shows growth

Enter your text here

What is the reading frequency?

Enter your text here

Antimicrobial susceptibility testing - broth dilution in microplates

How do you prepare the isolates to work with ?

e.g. colony picking, filter cloning…

Enter your text here

How do you preserve the strains for AST?

- ◯ in broth with glycerol
- ◯ in broth without glycerol
- ◯ Other

please specify

Do you calibrate isolates (quantification) before AST assay ?

- ◯ Yes
- ◯ No

What is the method used for strain calibration ?

Enter your text here

Antimicrobial susceptibility testing - broth dilution in microplates

Assay controls :

What control do you usually include in agar dilution assay ?

- ☐ Positive control : known strain without antimicrobial
- ☐ Positive control : strain with antimicrobial and an expected MIC profile
- ☐ Negative control : sterile media
- ☐ No controls
- ☐ Other

Please specify

Please specify genus/species of the control strains used

Enter your text here

Reading :

What is your reading method ?

- ☐ colony counting using magnifier (manual)
- ☐ automatized colony counting
- ☐ Other

please specify

Previous | Next

Antimicrobial susceptibility testing - broth dilution in microplates

Validation of the results and interpretation :

How do you validate the results obtained (control results excluded) ?

e.g. colony counts, CCU,...

Enter your text here

What is the number of repetition(s) used to validate a MIC value (replicates in a same run or independent replicates in different run):

Enter your text here

Please indicate the interpretation cut-off used if any :

Enter your text here

Do you have any further comments on agar dilution method ?

Enter your text here

Antimicrobial susceptibility testing - broth dilution in tubes

What is the medium used for broth dilution method in tubes?

Enter your text here

What is the antimicrobial concentration range used ?

- [ ] Full range
- [ ] truncated range (only several concentrations tested)

Incubation temperature :

Enter your text here

Incubation atmosphere :

Enter your text here

What is the incubation period?

please include start and end of reading if different, expressed in days if possible or indicate the reference used to start/ end the reading e.g. when the growth control shows growth

Enter your text here

What is the reading frequency?

Enter your text here

Antimicrobial susceptibility testing - broth dilution in tubes

How do you prepare the isolates to work with ?

e.g. colony picking, filter cloning…

Enter your text here

How do you preserve the strains for AST?

- ◯ in broth with glycerol
- ◯ in broth without glycerol
- ◯ Other

please specify

Do you calibrate isolates (quantification) before AST assay ?

- ◯ Yes
- ◯ No

What is the method used for strain calibration ?

Enter your text here

Antimicrobial susceptibility testing - broth dilution in tubes

Assay controls :

What control do you usually include in agar dilution assay ?

- [ ] Positive control : known strain without antimicrobial
- [ ] Positive control : strain with antimicrobial and an expected MIC profile
- [ ] Negative control : sterile media
- [ ] No controls
- [ ] Other

Please specify

Please specify genus/species of the control strains used

Enter your text here

Reading :

What is your reading method ?

- [ ] colony counting using magnifier (manual)
- [ ] automatized colony counting
- [ ] Other

please specify

Antimicrobial susceptibility testing - broth dilution in tubes

Validation of the results and interpretation :

How do you validate the results obtained (control results excluded) ?

e.g. colony counts, CCU,...

Enter your text here

What is the number of repetition(s) used to validate a MIC value (replicates in a same run or independent replicates in different run):

Enter your text here

Please indicate the interpretation cut-off used if any :

Enter your text here

Do you have any further comments on broth dilution method ?

Enter your text here

Antimicrobial susceptibility testing - other method

What is the medium used for this other method ?

Enter your text here

What is the antimicrobial concentration range used ?

- [ ] Full range
- [ ] truncated range (only several concentrations tested)

Incubation temperature :

Enter your text here

Incubation atmosphere :

Enter your text here

What is the incubation period?

please include start and end of reading if different, expressed in days if possible or indicate the reference used to start/ end the reading e.g. when the growth control shows growth

Enter your text here

What is the reading frequency?

Enter your text here

Antimicrobial susceptibility testing - other method

How do you prepare the isolates to work with ?

e.g. colony picking, filter cloning…

Enter your text here

How do you preserve the strains for AST?

- ◯ in broth with glycerol
- ◯ in broth without glycerol
- ◯ Other

please specify

Do you calibrate isolates (quantification) before AST assay ?

- ◯ Yes
- ◯ No

What is the method used for strain calibration ?

Enter your text here

Previous Next

Antimicrobial susceptibility testing - other method

Assay controls :

What control do you usually include in agar dilution assay ?

- [ ] Positive control : known strain without antimicrobial
- [ ] Positive control : strain with antimicrobial and an expected MIC profile
- [ ] Negative control : sterile media
- [ ] No controls
- [ ] Other

Please specify

Please specify genus/species of the control strains used

Enter your text here

Reading :

What is your reading method ?

- [ ] colony counting using magnifier (manual)
- [ ] automatized colony counting
- [ ] Other

please specify

Antimicrobial susceptibility testing - other method

Validation of the results and interpretation :

How do you validate the results obtained (control results excluded) ?

e.g. colony counts, CCU,...

Enter your text here

What is the number of repetition(s) used to validate a MIC value (replicates in a same run or independent replicates in different run):

Enter your text here

Please indicate the interpretation cut-off used if any :

Enter your text here

Do you have any further comments on this method ?

Enter your text here

Antimicrobial susceptibility testing - molecular / genomic approaches

Do you perform molecular or genomic approaches to determine AST ?

- ◯ Yes
- ◯ No

Antimicrobial susceptibility testing - molecular / genomic approaches

Please answer for one method at a time.

Name of the method used :

Enter your text here

Type of method :

- ◯ PCR based
- ◯ Genomic
- ◯ Other

Please specify

Please provide a short description of other method used or a reference article :

Enter your text here

Previous Next

Antimicrobial susceptibility testing - molecular / genomic approaches

Publication status of the method :

- ◯ Published
- ◯ Submitted
- ◯ None

Please provide a short description of the preliminary steps of the method :

e.g. DNA/library preparation

Enter your text here

What controls do you include in the assay?

Enter your text here

Please provide a short description of results analysis :

Enter your text here

Previous Next

Antimicrobial susceptibility testing - molecular / genomic approaches

Is another molecular/genomic method used in your laboratory?

- ◯ Yes
- ◯ No

Antimicrobial susceptibility testing - molecular / genomic approaches

Name of the method used :

Enter your text here

Type of method :

- ◯ PCR based
- ◯ Genomic
- ◯ Other

Please specify

Please provide a short description of other method used or a reference article :

Enter your text here

Previous | Next

Antimicrobial susceptibility testing - molecular / genomic approaches

Publication status of the method :

- ◯ Published
- ◯ Submitted
- ◯ None

Please provide a short description of the preliminary steps of the method :

e.g. DNA/library preparation

Enter your text here

What controls do you include in the assay?

Enter your text here

Please provide a short description of results analysis :

Enter your text here

Previous Next

Antimicrobial susceptibility testing - molecular / genomic approaches

Is another molecular/genomic method used in your laboratory?

- ◯ Yes
- ◯ No

Previous Next

Antimicrobial susceptibility testing - molecular / genomic approaches

Name of the method used :

Enter your text here

Type of method :

- ◯ PCR based
- ◯ Genomic
- ◯ Other

Please specify

Please provide a short description of other method used or a reference article :

Enter your text here

Previous Next

Antimicrobial susceptibility testing - molecular / genomic approaches

Publication status of the method :

- ◯ Published
- ◯ Submitted
- ◯ None

Please provide a short description of the preliminary steps of the method :

e.g. DNA/library preparation

Enter your text here

What controls do you include in the assay?

Enter your text here

Please provide a short description of results analysis :

Enter your text here

Previous Next

Antimicrobial susceptibility testing - available data

Do you have AST results for this mycoplasma species ?

- ○ Yes
- ○ No

Which method(s) was used to obtain these data ?

- ☐ Agar dilution method
- ☐ Broth dilution in tubes
- ☐ Broth dilution in microplates
- ☐ Other

Please specify

Please upload the completed table with data details

Add a document

Previous Next

Antimicrobial susceptibility testing - available data

Publication status :

◯ Yes

◯ Non

Please provide doi reference :

Enter your text here

Please indicate any intellectual property rights regarding this information or access to raw data

Enter your text here

Previous | Next

Antimicrobial susceptibility testing - available data

Are there strain(s) available in your archive for further MIC determination ?

- ◯ Yes
- ◯ No

Please upload the completed table with data details

Add a document

Please indicate any intellectual property rights regarding this information or access to raw data

Enter your text here

Previous   Next

Antimicrobial susceptibility testing - available strain(s) for sharing as control materials

For future work, mycoplasma isolates with known MIC will be required for sharing among the partners.

Are you willing to share some isolates on that purpose ?

- ◯ Yes
- ◯ No

Please give a short description of isolates you would be willing to share?

Enter your text here

Please indicate any intellectual property rights regarding this/these strain(s)

Enter your text here

Previous Next

Conclusion

Do you have any other comments before saving your answer?

Enter your text here

Now, please save your answer using the button down right.

We thank you for participating in this questionnaire!

If you have any further questions regarding this questionnaire, please contact the WP1 co-leaders Maryne Jaÿ and Sara Klose.

Should you have any other comments, feel free to contact the MyMIC animation team at mymic_animation@anses.fr.

Previous

Save
